# MUC1* Ligand, NM23-H1, Is a Novel Growth Factor That Maintains Human Stem Cells in a More Naïve State

**DOI:** 10.1371/journal.pone.0058601

**Published:** 2013-03-07

**Authors:** Benoit J. Smagghe, Andrew K. Stewart, Mark G. Carter, Laura M. Shelton, Kyle J. Bernier, Eric J. Hartman, Amy K. Calhoun, Vasilios M. Hatziioannou, Gabriele Lillacci, Brian A. Kirk, Brian A. DiNardo, Kenneth S. Kosik, Cynthia Bamdad

**Affiliations:** 1 Minerva Biotechnologies, Waltham, Massachusetts, United States of America; 2 The Neuroscience Research Institute and the Department of Molecular, Cellular and Developmental Biology, University of California Santa Barbara, Santa Barbara, California, United States of America; 3 Department of Mechanical Engineering, University of California Santa Barbara, Santa Barbara, California, United States of America; University of Cincinnati, United States of America

## Abstract

We report that a single growth factor, NM23-H1, enables serial passaging of both human ES and iPS cells in the absence of feeder cells, their conditioned media or bFGF in a fully defined xeno-free media on a novel defined, xeno-free surface. Stem cells cultured in this system show a gene expression pattern indicative of a more “naïve” state than stem cells grown in bFGF-based media. NM23-H1 and MUC1* growth factor receptor cooperate to control stem cell self-replication. By manipulating the multimerization state of NM23-H1, we override the stem cell's inherent programming that turns off pluripotency and trick the cells into continuously replicating as pluripotent stem cells. Dimeric NM23-H1 binds to and dimerizes the extra cellular domain of the MUC1* transmembrane receptor which stimulates growth and promotes pluripotency. Inhibition of the NM23-H1/MUC1* interaction accelerates differentiation and causes a spike in miR-145 expression which signals a cell's exit from pluripotency.

## Introduction

Both embryonic stem (ES) and induced pluripotent stem (iPS) cells hold great promise for the treatment of a wide variety of acquired or hereditary diseases [1], [2]. The major obstacles to clinical applications are: 1) developing cell culture methods that will comply with expected FDA requirements [3], [4]; 2) culturing enough high quality pluripotent stem cells; and then 3) directing them to differentiate into functional adult cells. FDA and European guidelines for human stem cell therapies require some version of Good Manufacturing Practice (GMP) for quality assurance and patient safety. Defining a GMP equivalent for stem cell therapies is challenging because these cells have traditionally been cultured in a milieu of largely undefined components, many of which are animal-derived [3]–[6]. Most protocols used today involve a supporting layer of fibroblast feeder cells [7], [8], their conditioned media [9] or Matrigel [10], [11]. These are complex mixtures of poorly characterized components that vary greatly from batch to batch, and therefore cannot be made GMP-compliant.

Several recent studies focused on the development of defined media that do not require feeder cells or their conditioned media. mTeSR and StemPro are two such defined media[5], however, they remain complex, having 18 and 14 different components, respectively, in addition to those in the base media. More recently, researchers reported a simpler bFGF-media, called E8, that is fully defined but apparently requires hypoxic conditions for growth [12]. Both mTeSR and E8 contain bFGF at 25-times the concentration at which it is normally used for stem cell culture. The use of bFGF at extremely high levels, in combination with a multitude of other growth factors, calls into question whether or not these media mimic stem cell growth *in vivo*, which could adversely affect attempts to direct differentiation to specific cell types.

Similarly, the quest for a defined substrate that supports stem cell adhesion and growth, without introducing biological or mechanobiological signals that alter stem cell fate, has been challenging [13], [14]. Extracellular matrix proteins as well as small molecule and polymer coatings have been tried [15]–[24]. However, many of these surfaces still require the use of feeder cell conditioned media, few have been shown to be effective for long-term growth of both ES and iPS cells and essentially all function by unknown mechanisms. Further, the effect of various surfaces on short term stem cell gene expression or long-term cell fate has not been adequately investigated.

To compound the problem, recent research indicates that human stem cells, cultured by standard methods are not truly pluripotent or “naïve”, rather they are in a more differentiated state called “primed” [25], [26]. Current methods for culturing human stem cells all depend on the addition of exogenous bFGF [27]–[30]. However, studies conclude that human stem cells in the naïve state cannot be maintained in bFGF [26]. Like murine primed cells, derived from the epiblast, conventional human ES cells grow as flattened colonies, are intolerant of passaging as single cells, have undergone X-inactivation and grow in bFGF by the TGF-beta/activin signaling pathway. In contrast, naïve stem cells grow in sheets or dome-shaped colonies, can be trypsinized and passaged as single cells at low densities, are less prone to spontaneous differentiation than traditional ES cells and grow by an as yet unidentified pathway. Thus far, researchers have only been able to temporarily revert human primed cells to the naïve state via ectopic expression of certain genes and treatment with kinase inhibitors [26].

The absence of the appropriate growth factor that maintains human stem cells in the naïve state could be the major reason behind the difficulties researchers encounter when working with human stem cells that are not encountered when working with mouse stem cells. In an attempt to create a growth system that enables self renewal of naïve stem cells, we discovered that NM23-H1 can be used as the only growth factor or cytokine required for the culture of human pluripotent stem cells. NM23-H1 was first identified as a tumor suppressor because of its reduced expression in metastatic cancer cells[31]. I-factor, which was isolated from a differentiation-resistant murine myeloid leukemia cell line and shown to be responsible for the inhibition of differentiation [32], was later identified as NM23-H1 [33]. In the subsequent years, other NM23s have been identified, H1 to 10, with H1 and H2 isoforms being the most studied to date. The NM23-H1 family of proteins is characterized by the presence of Nucleoside Diphosphae Kinase (NDPK) domains [34], [35], although its role in differentiation was shown to be independent of its catalytic function [36]. There have been a number of research articles published that report conflicting, in fact opposite results, regarding the function of NM23-H1 [37]. Reports that NM23-H1 is a differentiation inducer are countered by an equal number that report it is a differentiation inhibitor. Similarly, NM23-H1 has been reported to be a tumor suppressor and a tumor enhancer. These apparently mutually exclusive results could be explained by the fact that researchers were unwittingly testing NM23-H1 in different multimerization states, which raised the possibility that the different multimers have different functions [38]–[40].

In an earlier study [41], we reported that a cleaved form of the MUC1 transmembrane receptor, called MUC1*, that had previously only been detected on cancer cells [42] is expressed on undifferentiated human embryonic stem cells and mediates their growth in an bFGF-independent manner. That study showed that ligand-induced dimerization of MUC1* using either a bivalent antibody or dimeric NM23-H1 was sufficient to support extended undifferentiated growth of hESCs under feeder-free conditions. However, in the earlier study, only embryonic stem cells were tested, they were cultured on Matrigel, serial passaging was not performed, the effect on karyotype stability was not examined and the cells' ability to differentiate was not determined. The present study includes iPS as well as ES cells, Matrigel is replaced by a monoclonal antibody surface, serial passaging is performed, karyotype stability is demonstrated, and the differentiation potential of NM23-H1 cultured stem cells is determined. The function of different NM23-H1 multimers is explored. More importantly, the possibility that NM23-H1 could stabilize genetically unmodified human stem cells in the naïve state is investigated.

Here we clearly demonstrate that NM23-H1 in dimeric form, but not the hexameric form, alone is sufficient to inhibit stem cell differentiation, to allow long-term growth and maintenance of pluripotency and, in combination with a newly defined surface, increases expression of naïve markers.

## Materials and Methods

### Stem Cells and Culture

Human ES cells, H9 (WiCell), BGO1V/hOG cells (Life Technologies), human iPS cells, FTD19 clone 42 and ESIMR90 clone 4, (Kosik Lab) were cultured at 37°C and 5% CO_2_ on mitomycin-C inactivated Hs27 human foreskin fibroblasts or MEFs (ATCC), Matrigel (BD Biosciences) per manufacturer's instructions, Vitronectin at (6.25 µg or 12.5 µg), or anti-MUC1* mab MN-C3 (12.5 µg/well) coated onto 6-well plates (Vita plates, ThermoFisher; or BD Falcon # 353046). ES cell culture “Minimal Media” (**MM**) consisted of DMEM/F12/GlutaMAX I with 20% Knockout Serum Replacement, 1% non-essential amino acids stock and 0.1 mM β-mercaptoethanol (all from Life Technologies).

NM23-H1 as growth factor was added to MM (8 nM). Fibroblast growth factor (bFGF, Peprotech) at 4 ng/ml and 50% MEF conditioned media was added if stem cells were grown on Matrigel. NM23-H1 concentrations were calculated using the theoretical molecular weight of a NM23-H1 monomer (19.2 kDa).

Cells were passaged either by manual dissection or by Trypsinization every 4–7 days at a ratio of at least 1:3 and medium was changed every 48 hours. In some cases a Rho kinase inhibitor (Y-27632, Calbiochem) was added for the first 48 hours.

### MEF and HS27 conditioned media depletion

NM23-H1 antibodies (C20 and NM301, Santa Cruz biotechnology) were coupled to an AminoLink resin according to the manufacturer recommendations (Pierce) for depletion of NM23-H1. The column was equilibrated with PBS pH 7.4 and the conditioned media was applied to the column and incubated with end over end mixing for 45 min at 4 °C. The column was washed and bound proteins were eluted with 0.1 M glycine pH 2.5 into a tube containing 1/10 of the fraction volume of 1 M Tris pH 8.0. The elution fractions were concentrated and the molar protein concentration (NM23-H1) was determined from the optical density at 280 nm using an extinction coefficient of 1.35 for 1 mg/mL and the theoretical molecular weight of a recombinant NM23-H1 monomer (19.2 kDa). NM23-H1 depletion from the media was also confirmed by Western blot (see Methods S1)

### Human NM23-H1 WT and S120G protein expression

NM23-H1 WT and S120G mutant were cloned into expression plasmids containing either a C-terminal histidine tag or a strep tag II and expressed, after IPTG induction, in E. coli (BL21 DE3, New England Biolabs). Proteins were purified by affinity chromatography (NiNTA, Qiagen or Streptactin, IBA). Where indicated, NM23-H1 S120G was denatured and refolded; a stable dimer population was then isolated using size exclusion chromatography.

### Anti-MUC1* Antibodies

Monoclonal antibodies (mabs) were produced by immunizing mice with a synthetic peptide (JPT) corresponding to the first forty-five (45) amino acids of the extracellular domain, GTINVHDVETQFNQYKTEAASPYNLTISDVSVSDVPFPFSAQSGA (“MUC1*_ecd_ peptide”). Hybridomas were created by fusion of spleen cells with SP2/0 myeloma cells and screened by ELISA using the synthetic MUC1*_ecd_ peptide (ProMab Biotechnologies).

### Surface Plasmon Resonance

3.8% NTA-Ni^++^ SAM-coated SPR chips were prepared as described previously [43] and experiments performed using a Biacore3000 instrument. Running buffer was 10 mM HEPES pH 7.5, 150 mM NaCl, 50 µM EDTA and 0.005% Tween20, Flow rate was 5 µL/min. The surface was activated by 1% NiSO4 and histidine-tagged MUC1*_ecd_ peptide was immobilized. NM23-H1 variants (64 nM) were injected to measure binding. Before the next cycle, the surface was regenerated. Each sensograms were corrected for binding in absence of NM23-H1 protein. The protein molar concentrations were calculated using the theoretical molecular weight of a recombinant NM23-H1 monomer (19.2 kDa).

### Gold nanoparticles assay

2.5% NTA-SAM coated nanoparticles (AuNPs) were generated as described earlier [44], activated with 0.005% NiSO_4_ for 5 min at room temperature and loaded with a MUC1* peptide (QFNQYKTEAASRYNLTISDVSVSDVPFPFSAQSGAHHHHHH). NM23-H1 variants bearing Strep-tag II (see Methods S1) were added to the nanoparticles and color change was photographed after 75 minutes.

### miR-145 expression quantification

Total RNA was extracted from the samples using the *mir*Vana™ kit (Applied Biosystem, P/N: AM1561) per manufacturer's instructions. For each total RNA sample, two cDNA samples were synthesizes using the TaqMan® MicroRNA Reverse Transcription Kit (Applied Biosystems, P/N: 4366596) and two different stem-loop primers specific for miR-145 and the small nuclear RNA U6B (RNU6B), which served as an endogenous control. Quantification of miR-145 and RNU6B in the cDNA samples was performed using TaqMan® MicroRNA Assays (Applied Biosystems, P/N: 4427975) per manufacturer's instructions. The real-time PCR data were analyzed using the comparative C_t_ method. The relative amount of miR-145 in each sample was obtained by computing the difference between the miR-145 C_t_ and the corresponding RNU6B C_t_ (ΔC_t_). A second normalization was performed by subtracting the smallest ΔC_t_ from all the others in the data set (ΔΔC_t_).

### Immunocytochemistry of H9 cells and iPS cells

Cells were grown on 4-well chamber slides (Lab-Tek, cat # 177437)to50–75% confluency, then fixed with 4% Paraformaldehyde, blocked with 1% Goat Serum (Jackson ImmunoResearch, Cat # 005-000-121), 1% Bovine Serum Albumin, 0.1% Tween in PBS pH 7.4 for 1 hour at room temperature. Cells were incubated overnight at 4°C with: anti Oct-3/4 (C-10), anti SSEA-4 (813–70), anti TRA-1-81 (TRA-1-80) and anti Nanog (H-155), all antibodies were from Santa Cruz biotechnology (1:100), for the pluripotency markers. Cells were incubated overnight at 4°C with one of the primary antibodies (1:100 dilution) from the human embryonic germ layer characterization kit (Millipore) or an anti tubulin beta-III antibody (Covance). Cells were incubated overnight at 4°C with Tri-Methyl-Histone H3 (Lys27) Antibody (1/1600, Cell Signaling Technology) for X-activation status. Cells were then incubated 1 hour at room temperature with one of the following secondary antibody: Cy3 conjugated goat anti mouse antibody (Jackson Immunoresearch), FITC conjugated goat anti rabbit antibody (Jackson immunoresearch) Alexa- Fluor 555 conjugated goat anti rabbit antibody (Life Technologies) or an AlexaFluor-647 conjugated goat anti rabbit antibody (Life Technologies) at 1:50 or 1:100 dilution. Cells were washed with PBS pH 7.4 and incubated for 5 minutes at -20°C with 100% methanol. The wells were dried under the fume hood and mounted with Vectashield with DAPI (Vector Laboratories, Cat # H-1200). The imaging was done with Olympus IX 71 inverted microscope using 4X, 10X and or 20X objectives.

### MUC1* antibody surface for ES and iPS cell culture

Varying concentrations of MN-C3 monoclonal anti-MUC1* antibody was adsorbed onto the surfaces of either a standard tissue culture plate or Vita plates (ThermoFisher) by diluting antibody to 125 µg/mL–3.25 µg/mL in PBS pH 7.4. 1 mL of antibody was added to per well and incubated overnight at 4°C or at room temperature for 3 hrs. Wells were rinsed in PBS pH 7.4 prior to use.

### Growth rate and doubling time measurement

For a daily growth rate of *r*: 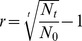 where *N_0_* is the number of cells seeded at time *t = *0, *N_t_* is the number of cells collected at time *t* (days), doubling time in hours (*T_d_*) is given by: 
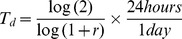


### Teratoma formation

ES cells serially passaged (p16) on a monoclonal anti-MUC1* antibody (MN-C3) surface in NM23-H1-MM (1.5 million/site in 30% matrigel) were injected in the kidney capsule and in the testis of mice (Foc Chase SCID-beige, male, 6weeks old from Charles River) for teratoma formation analysis. Tumors were fixed over night, embedded in paraffin, cut into 5 µm serial sections and stained (Hematoxylin and eosin) to detect embryonic germ cell layers (endoderm, mesoderm and ectoderm). The teratoma formation and analysis was done by Applied Stem Cell (Menlo Park, CA).

### Real Time PCR

Total RNA was extracted from the samples using TRIzol^®^ Reagent (Life Technologies) per manufacturer's instructions and treated with TURBO DNA-*free*™ kit (Life Technologies). Quantification of FoxA2 (Applied Biosystems, Assay ID: Hs00232764_m1), XIST (Applied biosystems Assay, ID: Hs01079824_m1), Otx2 (Applied biosystems, Assay ID: Hs00222238_m1), Lhx2 (Applied Biosystems, Assay: Hs00180351_m1), Klf2 (Applied Biosystems, Assay ID: Hs00360439_g1), Klf4 (Applied Biosystems, Assay ID:Hs00358836_m1), Nanog (Applied Biosystems, Assay ID: Hs02387400_g1), Oct4 (POU class 5 homeobox 1) (ABI assay ID Hs007742896_s1) and GAPDH (Applied Biosystems, P/N: 4310884E), in the RNA samples was performed using TaqMan® One Step RT-PCR Master Mix Reagents (Applied Biosystems, P/N: 4309169) per manufacturer's instructions. For Figure 5i, cDNA was first generated with Random Hexamers (Life Technologies) using Super Script II (Life Technologies) and subsequently assayed for the above genes using TaqMan® Gene expression Master Mix (P/N 4369016). The real-time PCR data were analyzed using the comparative C_t_ method. The relative amount of each transcript in each sample was obtained by computing the difference between the target C_t_ and the corresponding GAPDH (ΔC_t_). A second normalization was performed by subtracting the MEF/bFGF sample ΔC_t_ from all the others in the data set (ΔΔC_t_).

## Results

### Fibroblast Conditioned Media Cannot Support Stem Cell Growth if Depleted of NM23-H1

**Figure 1:**
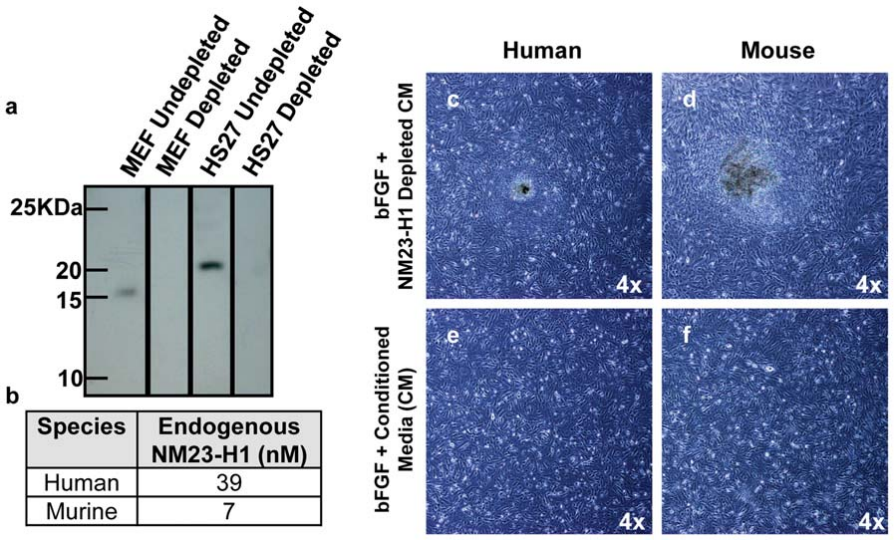
Conditioned media depleted of NM23-H1 no longer supports pluripotent stem cell growth. **a**) Conditioned media from either murine (MEFs) or human (HS27) embryonic fibroblast cells was immuno-depleted of NM23-H1. Western blot confirmed that the depleted media no longer contains NM23-H1. **b**) NM23-H1 was captured by affinity chromatography, eluted and its concentration quantified by the optical density at 280nm. Human conditioned media contains 5-times more NM23-H1 than murine conditioned media. Human ES cells on Matrigel were cultured with: **c, d**) NM23-H1-depleted media, **e, f**) bFGF plus complete conditioned media. NM23-H1-depleted conditioned media induced differentiation (**c, d**).

Our previous work showed that ligand-induced dimerization of the MUC1* growth factor receptor by NM23-H1 [31] supported human stem cell growth [41]. Since fibroblast feeder cells, or their conditioned media, are widely used for the growth of ES and iPS cells, we investigated whether feeder cells were merely providing stem cells with NM23-H1.NM23-H1 was immuno-depleted from fibroblast feeder cell conditioned media (Fig. 1a, b) then tested for the ability to support stem cell growth. Stem cells cultured in bFGF and the NM23-H1-depleted conditioned media either did not proliferate at all or prematurely differentiated (Fig. 1c, d) compared to stem cells cultured in bFGF and the conditioned media (Fig 1e, f). This raised the question of why conditioned media plus bFGF are required if we previously showed that NM23-H1 alone is sufficient. However, in our previous study a mutant form of NM23-H1 (S120G) that prefers dimerization was used. We therefore examined the effect of different NM23-H1 mutlimers on differentiation and on their ability to bind to their cognate ligand, MUC1*.

### NM23-H1 characterization and competitive inhibition of the NM23-H1-MUC1* interaction

We discovered that the key to NM23-H1 function is its multimerization state. NM23-H1 can exist as a monomer, dimer, tetramer or hexamer, depending on its concentration, sequence and method of expression [45], [46]. We previously showed that NM23-H1 is a ligand of MUC1* and that the MUC1* growth factor receptor is activated by ligand-induced dimerization of its extra cellular domain [42]. It therefore follows that the dimeric form of NM23-H1 should be the multimer that promotes stem cell growth and pluripotency. Notably, NM23-H1 mutants that prefer dimer formation, such as NM23-H1 S120G, have been isolated from cancers, which are cells that have lost their ability to limit stem-like self-replication [45], [47].

**Figure 2:**
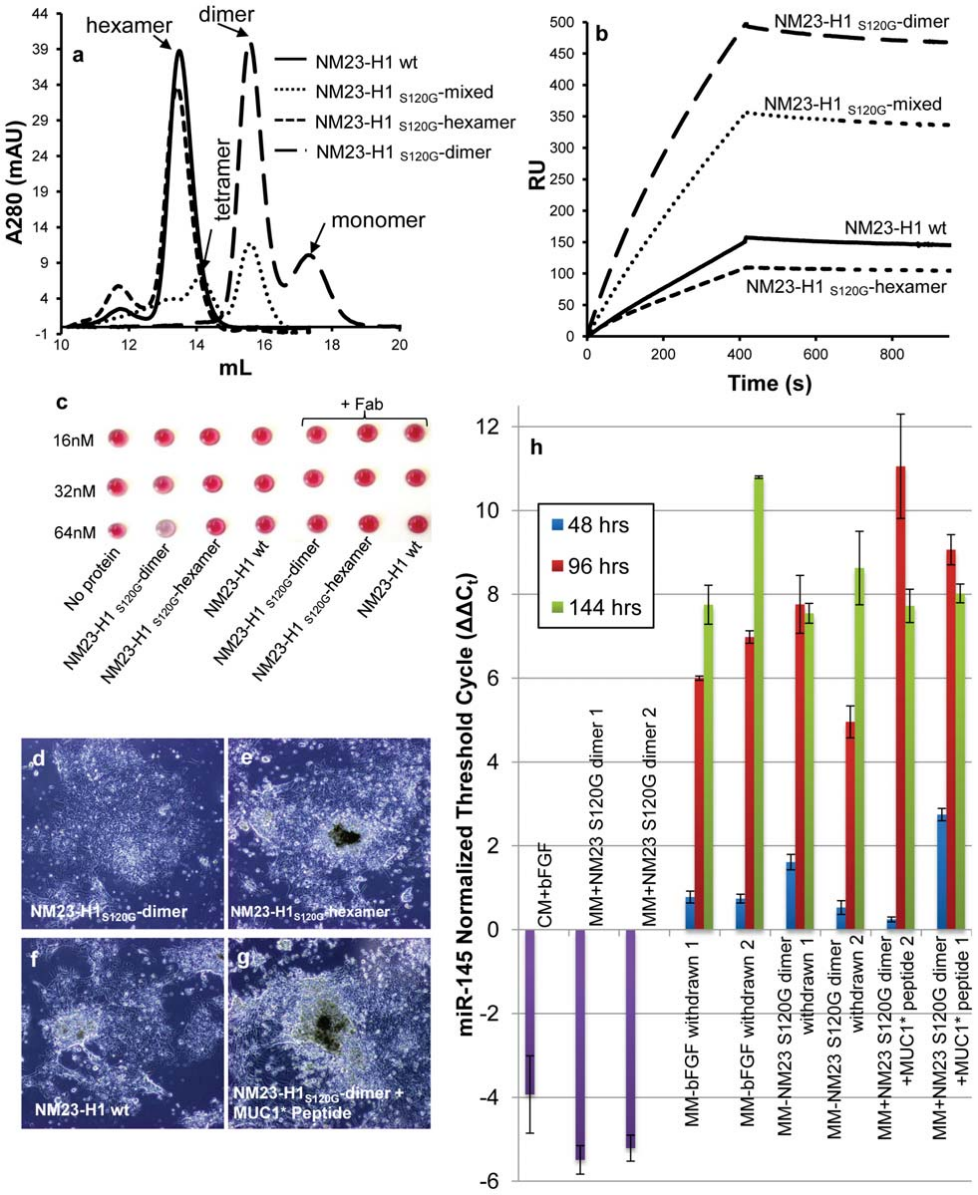
Different NM23-H1 multimers are generated and assayed for function. **a**) Recombinant NM23-H1 wt (wild type) and NM23-H1_S120G_ mutant were expressed using different protocols that resulted in the formation of different multimerization states, which were characterized and then purified by size exclusion chromatography. Simple expression and collection of the soluble protein of NM23-H1 -wt and NM23-H1_S120G_ mutant results in a population that is essentially all hexamer. Denaturation and refolding of the NM23-H1_S120G_ produces a stable dimer population. A mixture of hexamers, tetramers and dimers was generated such that it contained ∼50% dimer, NM23-H1_S120G_-mixed. **b**) NM23-H1_S120G_ or wild type multimers were tested by Surface Plasmon Resonance (SPR) to determine their ability to bind to a synthetic MUC1* extra cellular domain (ecd) peptide immobilized on the chip surface. The amount of NM23-H1 binding to the MUC1* peptide corresponds to the concentration of dimer present in each sample. **c**) Nanoparticles presenting the MUC1*_ecd_ peptide were mixed with NM23-H1-wt, NM23-H1_S120G_-dimer or NM23-H1_S120G_-hexamer containing the Strep-tag II. A nanoparticle color change from pink to blue/gray indicates binding. NM23-H1 dimer binds to the MUC1*_ecd_ peptide at 64nM while the hexamer, whether wild type or S120G mutant, does not. The interaction was competitively inhibited by an anti-MUC1* Fab, showing that the color change was due to the specific interaction between NM23-H1dimers and MUC1*_ecd_. **d, g**) H9 hES cells on Matrigel were cultured in: **d**) NM23-H1_S120G_-dimer, **e**) NM23-H1_S120G_-hexamer, **f**) NM23-H1-wt or **g**) NM23-H1_S120G_-dimer plus a synthetic MUC1*_ecd_ peptide (1 µM). Only NM23-H1_S120G_-dimers supported pluripotent stem cell growth. Hexamers or inhibition of the NM23-H1_S120G_-dimer-MUC1* interaction resulted in immediate differentiation. All images 4X. **h**) H9 hES cells were cultured in either bFGF plus conditioned media or in NM23-H1_S120G_-dimer, and then allowed to differentiate by withholding the growth factor. Some cells cultured in NM23-H1_S120G_-dimer continued to receive the growth factor but also received the MUC1*_ecd_ peptide (1 µM) to competitively inhibit the NM23-H1-MUC1* interaction. miR-145, a marker for exit from pluripotency, is measured by RT-PCR as a function of time. Competitive inhibition of the NM23-H1-MUC1* interaction caused an earlier spike in miR-145 than that by merely withholding the growth factor, demonstrating that interrupting the NM23-H1-MUC1* interaction induces differentiation.

In order to test the function of various NM23-H1 multimerization states, we produced NM23-H1 wt and the S120G mutant in different multimerization states. NM23-H1-wt exists primarily as hexamers. S120G can exist as a hexamer or a dimer, depending on the expression and purification protocol. Analysis by size exclusion chromatography (SEC), native gel, and Western blot showed that simply collecting the soluble fraction of NM23-H1 S120G results in a population that is predominantly hexameric (NM23-H1_S120G_-hexamer). However, denaturing and refolding NM23_S120G_ produces a population consisting primarily of dimer (Fig. 2a and Fig. S1a, b).

We tested the ability of NM23-H1 hexamers and dimers to bind to the MUC1*_ecd_ peptide in a direct binding assay using Surface Plasmon Resonance (SPR) [48]. A synthetic MUC1*_ecd_ peptide was immobilized onto a gold chip [49]. NM23-H1-wt, NM23-H1_S120G_-dimer, NM23-H1_S120G_-hexamer, or a sample containing 50% of NM23-H1_S120G_ dimer were separately flowed over the peptide surface. The amount of NM23-H1 that bound to the peptide surface was a function of the amount of dimer present in each sample (Fig. 2b). NM23-H1_S120G_-dimer showed robust binding to the immobilized MUC1* peptide, while NM23-H1 -wt and NM23-H1_S120G_-hexamer, which are mainly hexamers, showed minimal binding. Note that the SPR signal is directly proportional to the mass of the molecular species bound at the solution-peptide surface interface [50]. Therefore, if the hexameric form of NM23-H1 bound to the MUC1* peptide surface, the greater mass of the hexamer should result in 3-times more resonance units (RUs) than the dimer. The fact that the amount of hexamer binding was minimal is consistent with the idea that NM23-H1 hexamer does not bind to the MUC1* receptor.

To further explore the binding characteristics of NM23-H1 dimers versus hexamers, we performed a nanoparticle assay in which the MUC1*_ecd_ peptide was affinity immobilized on SAM-coated gold colloids [44]. For this assay, all NM23-H1 variants were expressed and purified with the Strep-tag II (Fig S2). The addition of NM23-H1_S120G_-dimer induced a pink to blue solution color change, indicating a specific binding to the MUC1* peptide that can be inhibited by the addition of an anti-MUC1* Fab. Conversely, the addition of NM23-H1-wt or NM23-H1_S120G_- hexamer did not induce a color change, consistent with the notion that the hexamer does not bind to the MUC1* receptor (Fig. 2c).

The ability of NM23-H1 dimers to support stem cell growth was tested by reconstituting the NM23-H1 immuno-depleted conditioned media (from fibroblast feeder cells) with NM23-H1_S120G_-dimer. The results show that in contrast to the depleted media, which induced differentiation, conditioned media that was reconstituted with NM23-H1 dimers supported undifferentiated stem cell growth that was indistinguishable from control cells. More importantly, we discovered that the addition of NM23-H1_S120G_-dimer eliminated the need for the addition of exogenous bFGF (Fig. S3). To further explore the function of the differentNM23-H1 multimers on stem cell pluripotency, we cultured human ES cells in bFGF-free minimal stem cell media (MM) containing either NM23-H1-wt, NM23-H1_S120G_-dimer or NM23-H1_S120G_-hexamer. Culturing ES cells in NM23-H1_S120G_-dimer, produced completely undifferentiated stem cells (Fig. 2d), but NM23-H1_S120G_-hexamer and NM23-H1-wt (mostly hexamers) rapidly differentiated (Fig. 2e and f). The addition of a synthetic MUC1*_ecd_ peptide to competitively inhibit interaction between NM23-H1_S120G_-dimer and MUC1* resulted in the highest degree of differentiation (Fig. 2g). Note that the concentration of NM23-H1_S120G_-dimer used in the nanoparticle assay was greater than the concentrations used in our stem cell media due to insensitivity of the nanoparticle assay. NM23-H1_S120G_-dimer was stable in the culture media over a period of 2 days (Fig. S4). These results demonstrate that it is the specific interaction of NM23-H1_S120G_-dimer with the extra cellular domain of the MUC1* growth factor receptor that promotes pluripotency.

### miR-145 spikes when NM23-H1-MUC1* interaction is inhibited

An increase in miR-145 expression signals the stem cells' exit from pluripotency [51]. RT-PCR measurements showed that inducing differentiation by withdrawing the growth factor (either bFGF or NM23-H1 _S120G_-dimer) stimulates expression of miR-145 with a peak at 144 hours. However, competitive inhibition of the NM23-H1_S120G_-dimer-MUC1* interaction by the free MUC1*_ecd_ peptide resulted in an earlier (96 hours) and larger spike in the expression of miR-145 (Fig. 2h). The level of expression of miR-145 indicates initiation of differentiation (Fig. S5). Non-synchronized and non-uniform differentiation within a well can give rise to differences. However, the two biological replicates analyzed show the same trend in miR-145 expression. These results confirm that inhibition of NM23-H1_S120G_-dimer -MUC1* interaction induces differentiation and that only dimeriztion of the MUC1* growth factor receptor's extra cellular domain by NM23-H1_S120G_-dimer promotes self-renewal of pluripotent stem cells.

### Performance of NM23-H1 Minimal Media Compared to bFGF-Based Media

**Figure 3:**
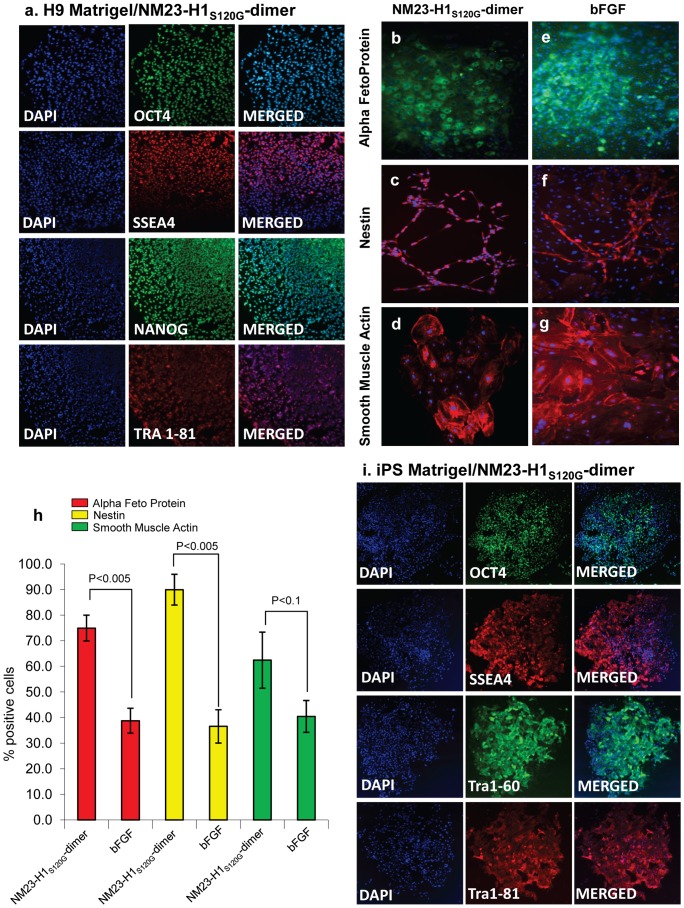
Human ES cells serially passaged in NM23-H1-MM express pluripotency markers, differentiate down all three germlines and display coordinated differentiation. H9 hES cells on Matrigel were cultured for at least six passages in either NM23-H1-MM (minimal stem cell media) or bFGF plus MEF conditioned media. a) NM23-H1-MM cultured cells stained positive for the presence of the typical pluripotency markers.. NM23-H1 cultured cells were allowed to differentiate by the embryoid body method (Methods S1) then stained with nuclear marker DAPI and antibodies against markers of the three germlines: b, e) endoderm - alpha feto protein, c, f) ectoderm - nestin, and d, g) mesoderm - smooth muscle actin. Cells cultured in either NM23-H1-MM or bFGF-CM both differentiated down all three germlines. Cells that had been cultured in NM23-H1-MM displayed apparently coordinated differentiation with most cells in a cluster differentiating down the same germline (b–d), whereas cells cultured in bFGF did not (e–g). h) The percentage of cells in each cluster that expressed the same germline marker was quantified (n = 5 to 7). Cells that had been cultured in NM23-H1-MM have a higher percentage of cells in the same cluster differentiating down the same germline than cells that had been cultured in bFGF. i) iPS cells on Matrigel, cultured for six passages in NM23-H1-MM stained positive for the presence of the typical pluripotency markers. All images 4X

Human ES cells, serially passaged in NM23-H1_S120G_-dimer in minimal stem cell media (NM23-H1-MM), grew comparably to cells cultured in bFGF on feeder cells or in bFGF with conditioned media on Matrigel (Fig. S6 a-d). Immunocytochemistry (ICC) confirmed that the cells grown in NM23-H1-MM expressed the typical pluripotency markers (Fig. 3a) and karyotype was normal (Fig. S7a). H9 human ES cells cultured in NM23-H1-MM were also able to differentiate down all three germlines. Cells were allowed to differentiate by the embryoid body method [52], [53] (supplemental method), then stained for three germline markers: alpha feto protein, smooth muscle actin and nestin. Both the stem cells cultured by standard methods in bFGF and stem cells cultured in NM23-H1-MM differentiated down all three germlines, but interestingly, in many cases most of the cells within a single embryoid body committed to the same cell fate (Fig. 3b-d). In contrast, stem cells grown by stimulating the FGF pathway do not exhibit this uniform differentiation; in each cell cluster, multiple nuclei do not express the marker that is being assayed for, indicating that neighboring cells are differentiating down a different germline (Fig. 3e-g). Quantitative analysis of embryoid body ICC images verified that the percentage of cells, within an embryoid body, that were differentiating down the same germline was higher when cells had been cultured in NM23-H1-MM than when they were cultured in bFGF (Fig. 3h).

iPS cells cultured in NM23-H1-MM grew faster than the control cells cultured in bFGF, requiring passage several days earlier than the control cells, and when on feeder cells formed larger, more well-defined colonies (Fig. S6 e, f). The iPS cells cultured in NM23-H1-MM grew as well on Matrigel as they had on the feeder cells (Fig. S6g) expressed the typical pluripotency markers (Fig. 3i), and had unchanged karyotype (Fig. S7b).

### Novel Stem Cell Growth Surface

**Figure 4:**
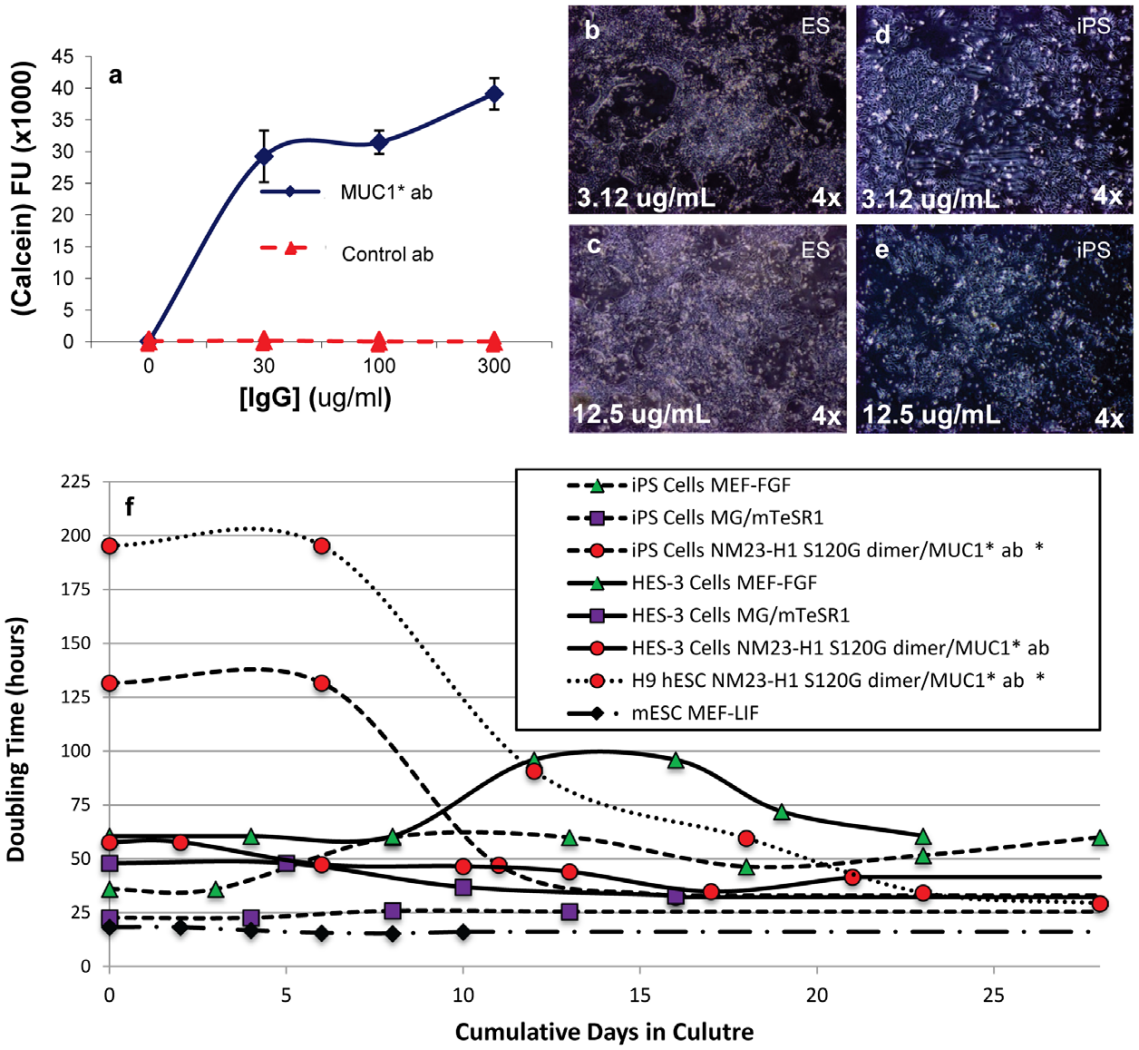
Anti-MUC1* antibodies are a novel surface coating that enables stem cell attachment and growth. **a**) An anti-MUC1* rabbit polyclonal antibody or a control IgG antibody were adsorbed at varying concentrations onto a tissue culture treated multi-well plate. BGO1V/hOG hES cells were plated onto the surfaces and allowed to grow for 48 hours. A Calcein AM assay to quantify cell number was performed. Anti-MUC1* antibody, but not the control antibody, enabled stem cell attachment and growth. BGO1V/hOG hES cells were cultured for 20 passages in NM23-H1-MM without a decrease in pluripotency or change in karyotype (data not shown). **b–e**) H9 hES cells (**b, c**) or iPS cells (**d, e**) were plated onto Vita_TM_ multi-well plates, reported to have higher protein binding capability, that were coated with either 3.12 µg/mL (**b, d**) or 12.5 µg/mL (**c, e**) of a monoclonal anti-MUC1* antibody, MN-C3. Cells attached and proliferated as a function of antibody concentration, with maximal attachment observed at 12.5 µg/mL. **f**) Doubling times forhuman ES cells (H9 or HES-3), human iPS cells and mouse ES cells were measured as a function of various culture media and surfaces. The traces marked by red circles/dotted line and red circles/dashed line (human H9 ES and iPS cells, respectively) show the change in doubling time as cells transition from bFGF-based media to NM23-H1 media on anti-MUC1* surface.

Because the biochemical nature of a surface can alter characteristics of stem cells, we developed a novel stem cell growth surface comprised only of components known to promote pluripotency. We reasoned that if ligand-induced dimerization of the MUC1* extra cellular domain is sufficient for stem cell growth and MUC1* is expressed on all undifferentiated stem cells [41], we could design a surface that heightens this interaction and eliminates the need for other factors that may introduce unwanted effects. We coated the surface of a multi-well cell culture plate with anti-MUC1*_ecd_ antibodies that function as both a method for stem cell attachment and for stimulating growth by dimerizing the MUC1* receptor. Stem cells attached to the surface as a function of antibody concentration, with maximal cell attachment reached at 30 µg/mL of antibody in the coating solution; essentially no stem cells attached to an identical surface coated with an irrelevant control antibody (Figure 4a). By switching to a plate with a high protein binding capacity surface (Vita, ThermoFisher), the concentration of antibody in the coating solution, required for maximal stem cell attachment was reduced by more than half to 12.5 µg/mL (Figure 4b–e). Antibody concentrations on plate surfaces were stable over the four day growth period, under typical stem cell growth conditions (data not shown). When stem cells were plated onto surfaces coated with a monoclonal anti-MUC1* antibody, proliferation was observed even when no growth factor was added into the media–presumably due to the dimerization of MUC1* from the surface-immobilized antibodies. However, growth rate was vastly improved by the use of NM23-H1 dimers in the minimal media (data not shown). In some cases, a Rho kinase inhibitor [54] was present during the first 48 hours, which increased attachment to the surfaces, but did not affect survival.

We next compared the growth rates of stem cells cultured in this new system to other more standard methods. Doubling time was measured for a number of human stem cells lines, when cultured in NM23-H1 on our antibody surface, bFGF on MEFs or mTeSR on Matrigel as well as for mouse ES cells cultured in LIF on MEFs. Because cells undergo a temporary decrease in growth rate when transitioning from bFGF-based media to NM23-H1 media, doubling times were measured for cells in transition as well as for steady state growth in NM23-H1 (Fig. 4f, red circles). Once cells transition to the NM23-H1 media, cells grew at about the same rate as cells cultured in mTeSR, which was almost twice as fast as cells cultured in bFGF on MEFs, but a bit slower than mouse ES cells (Fig. 4f).

**Figure 5:**
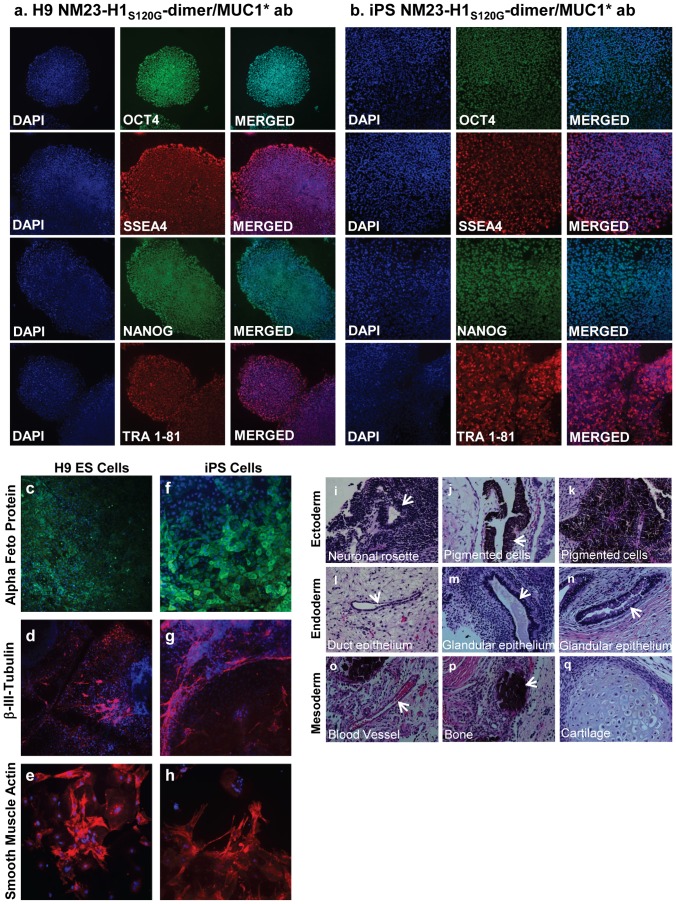
ES and iPS cells cultured long-term in NM23-H1-MM on Anti-MUC1* surfaces express pluripotency markers and differentiate down all three germlines. a, b) H9 ES cells and iPS cells serially passaged on a monoclonal anti-MUC1* antibody surface in NM23-H1-MM stained positive for typical pluripotency markers. c, h) After more than 20 passages (H9, P20; iPS, P28), cells were allowed to differentiate by embryoid body method (see Methods S1). Staining with nuclear marker DAPI and antibodies against markers of the three germlines, endoderm - alpha feto protein (c, f), ectoderm–beta-III-tubulin (d, g), and mesoderm - smooth muscle actin (e, h) shows that the cells differentiate normally down all three germlines. (All images 4X). i–q) ES cells serially passaged (p10) on a monoclonal anti-MUC1* antibody surface in NM23-H1-MM were injected in the kidney capsule and in the testis of mice for teratoma formation analysis (Applied Stem Cell, Menlo Park, CA). Tumors were fixed, embedded in paraffin, cut into sections and stained (Hematoxylin and eosin) to detect embryonic germ cell layers (endoderm, mesoderm and ectoderm). Typical structures from each germ layer were detected. All images x200.

After 20 passages in NM23-H1-MM on anti-MUC1* antibody surfaces, measurement of pluripotency genes by immunocytochemistry (ICC) (Fig. 5 a, b) and FACS analysis (Fig. S8 a–d) confirmed that human stem cells (ES and iPS) cultured with our system were pluripotent. The stem cells cultured in NM23-H1 on anti-MUC1* surfaces had unchanged karyotype after more than 28 passages (Fig. S7c, d). ES and iPS cells, after passage 20 and passage 28 respectively, differentiated down all three germlines (Fig. 5c–h). As a further demonstration of pluripotency, stem cells cultured in NM23-H1 over anti-MUC1* antibody surfaces readily formed teratomas that showed characteristics of all three germlines (Fig. 5i–q).

To assess the general applicability of our defined stem cell growth system, we compared the expression levels of pluripotency genes and miR-145, an indicator of the cell's exit from pluripotency, for several stem cells lines (iPS, H14, H7 and H9) that were cultured in either NM23-H1-MM on anti-MUC1* antibody surfaces or in bFGF on MEF feeder cells for 10–12 passages. Stem cells cultured in NM23-H1-MM on anti-MUC1* antibody surfaces express essentially the same or higher levels of the pluripotency genes than cells cultured in bFGF on MEFs (Fig. S9). These results clearly indicate that our system not only allows the maintenance of self renewal but, more importantly, promotes pluripotency.

### Naïve or Primed

**Figure 6:**
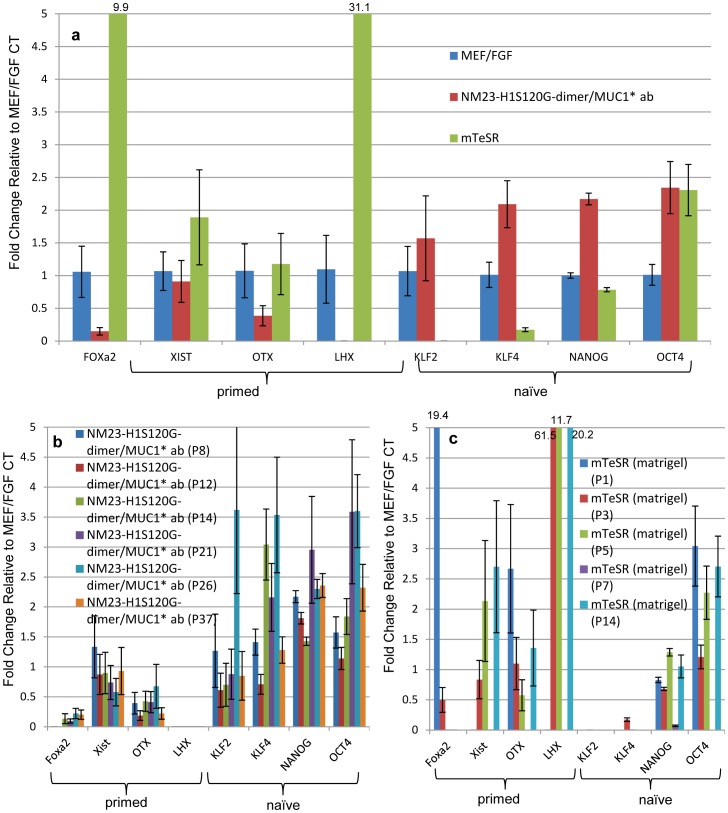
Human stem cells cultured in NM23-H1-MM over anti-MUC1* antibody surfaces express higher levels of naïve markers and lower levels of primed markers. RT-PCR was used to quantify expression of a subset of naïve markers that included Oct4, Nanog, Klf4 and Klf2, which should be high in the naïve state, and a subset of primed markers that included FoxA2, XIST, Otx2 and Lhx2, which are high in the primed state but low in the naïve state. Measurements were normalized to housekeeping gene GAPDH and expressed as fold change to H9 ES cells cultured in 4ng/ml bFGF over MEFs (control, n = 3). a) H9 ES cells cultured in NM23-H1-MM on anti-MUC1* antibody (MN-C3) surfaces, on average, showed increased expression of naïve markers and decreased expression of primed markers (n = 6). Conversely, H9 cells cultured in mTeSR over Matrigel showed decreased expression of naïve markers and increased expression of primed markers (n = 4). b) Individual measurements of the subset of naïve or primed markers are plotted as a function of passage number for NM23-H1-MM over anti-MUC1* antibody surfaces and c) for mTeSR over Matrigel. The trend toward the naïve state increased with successive passage in NM23-H1-MM but not with mTeSR. Large standard error for some experiments may be due to contamination of visually pluripotent stem cells with newly differentiating stem cells. For statistical analysis see Fig. S10 and S11.

To further assess the quality of stem cells cultured in NM23-H1-MM on anti-MUC1* antibody surfaces, we measured expression levels of a subset of genes that are thought to be indicators of human stem cells being in the “naïve” or ground state. In addition to Oct4 and Nanog, Klf4 and Klf2 are usually high in naïve stem cells, while FoxA2, XIST (an indicator of chromosome X-inactivation), Otx2 and Lhx2 are very low or not expressed. The reverse pattern of gene expression happens when cells are in the “primed” state, which is thought to be a more committed state. We compared expression levels of these genes in stem cells that were cultured in either NM23-H1-MM on anti-MUC1* antibody surfaces, bFGF on MEF feeder cells or mTeSR on Matrigel. Stem cells cultured in NM23-H1-MM on anti-MUC1* antibody surfaces expressed higher levels of naïve markers and lower levels of primed markers compared to cells cultured in bFGF on MEFs or in mTeSR on Matrigel (Fig. 6a). Cells cultured in mTeSR on Matrigel expressed higher levels of the genes that are indicators of the primed state, and lower levels of most of the naïve markers than cells cultured in bFGF over MEFs (Fig. 6a). A statistical analysis shows that the differences in gene expression between our system and other culture conditions are highly significant (Fig. S10). With successive passage number, a trend toward the naïve state was noted when stem cells were cultured in NM23-H1-MM (Fig. 6b). A statistical analysis shows that the correlation between increase of naïve markers expression and passage number is highly significant (P<0.0001, Fig. S11a). However, no significance was observed for the expression of the primed markers over the passage number (P = 0.482, Fig. S11a). Similar analysis of stem cells cultured in mTeSR on Matrigel did not show any correlation between gene expression and passage number (Fig. 6c, Fig. S11b). Although stem cells that were harvested for RT-PCR quantification were essentially 100% pluripotent by visual inspection based on cell morphology and nucleus to cytoplasm ratio, it is possible that large standard errors or lack of an apparent trend is due to having a cell population that contained some newly differentiating cells. To assess the contribution of surface alone, we plated ES cells that had been growing for 45 passages in bFGF on MEFs onto a layer of recombinant Vitronectin. The cells were then cultured in either NM23-H1-MM, bFGF plus MEF conditioned media or mTeSR for a single passage then assayed for expression of a subset of the naïve and primed markers. Although cells cultured in NM23-H1-MM showed higher expression of the naïve markers and lower expression of the primed markers compared to either bFGF or mTeSR, growth on Vitronectin resulted in decreased expression of naïve markers and increased expression of primed markers for all the media tested (Fig. S12). These results imply that growth on Vitronectin tends to prime stem cells.

**Figure 7:**
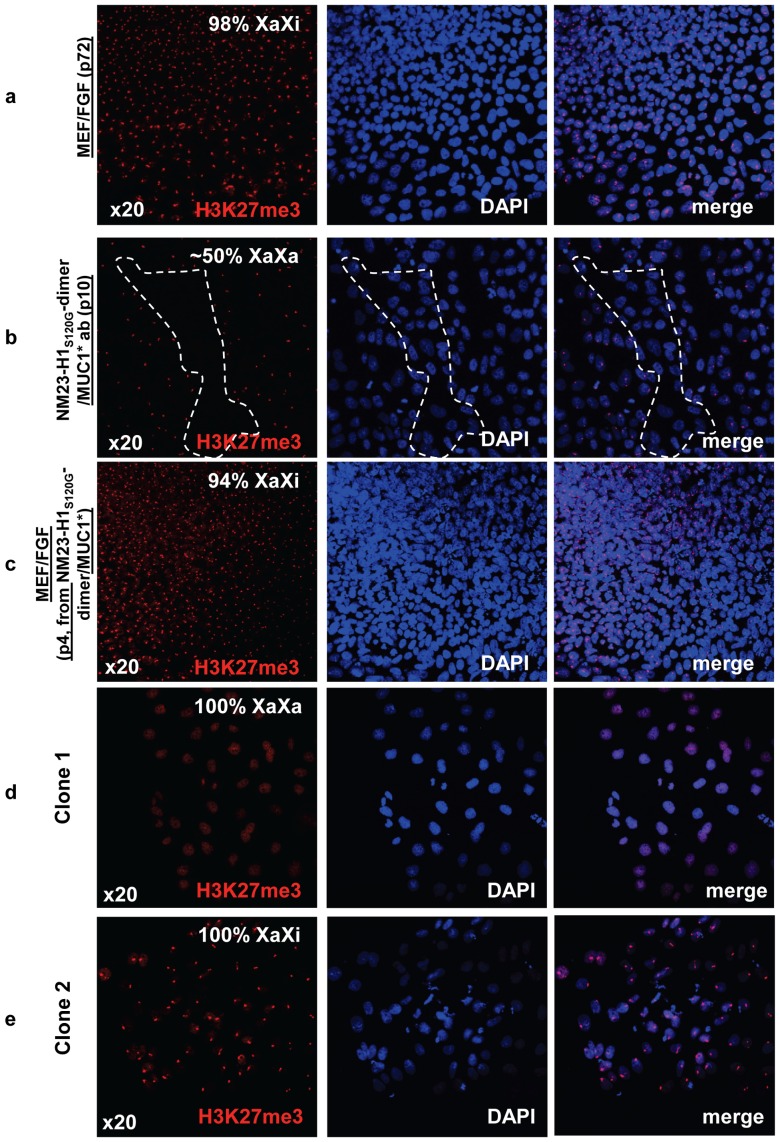
hES cells cultured in NM23-H1-MM on Anti-MUC1* surfaces are pre X-inactivation, characteristic of naïve state. **a**) Staining with nuclear marker DAPI and antibodies against tri-methylated (Lys27) Histone 3 shows that ES cells cultured in bFGF over MEFs are XaXi or post X-inactivation, characteristic of primed state. **b**) The cells shown in (a) were then transferred to anti-MUC1* antibody surfaces and cultured in NM23-H1 media for 10 passages then stained for X-activation status as in (a). The confocal images show a 50/50 mix of regions that are XaXa (dotted region) and others that are XaXi. **c**) The ES cells shown in (b) were then transferred back again to culture in bFGF over MEFs for 4 passages and images show 95% reversion to the XaXi, characteristic of primed state. **d, e**) ES cells cultured in NM23-H1 media over an anti-MUC1* antibody surface for 14 passages were serially diluted and allowed to grow until isolated colonies were observed. Cells were stained with nuclear marker DAPI and antibodies against tri-methylated (Lys27) Histone 3 to measure Chromosome-X status. X-activation status was clonal as we isolated clones with 100% X-inactivated (XaXi) and clones with 100% X-activated (XaXa).

To further investigate the naïve versus primed state of human stem cells cultured in NM23-H1 media on antibody surfaces, we looked at the pattern of a specific histone modification. Female stem cells in the primed state have already undergone X-inactivation (XaXi), whereas cells in the naïve or true ground state cells have two active X chromosomes (XaXa) [25]. One way to measure the X-activation state is to measure expression levels of the XIST gene, which is a non-translated RNA transcript that binds to the chromatin of the inactive X-chromosome and can be elevated when X is inactivated. However, a more stringent method is to visualize the expression pattern of tri-methylated (Lysine 27) histone 3 (“H3K27me3"). ICC staining of H3K27me3, stains the entire genome at a low level that has been described as a weakly stained “cloud”, while the condensed inactive X chromosome, of the primed state, makes H3K27me3staining appear as a discrete spot [26]. We measured the change in the pattern of H3K27me3 as we transitioned human ES cells from culture in bFGF over MEFs to NM23-H1-MM over an anti-MUC1* antibody surface, then back again to culture in bFGF over MEFs. Human stem cells cultured in bFGF were 100% in the inactive X state, indicated by the condensed dot pattern of H3K27me3 staining (Fig. 7a). In stark contrast, after ten passages in NM23-H1-MM over anti-MUC1* antibody surface, cells were about a 50/50 mix of cells with active X and inactive X (Fig. 7b). When the cells were returned to culture in bFGF, ∼85% of the cells showed X-inactivation by the second passage and 94% by the fourth passage (Fig. 7c). When stem cells were transitioned from culture in bFGF to culture in NM23-H1-MM, the percentage of cells with two active X chromosomes increased with passage number. By passage eight, ∼50% of the stem cell population had reverted to the XaXa state (data not shown). We observed that the distribution of cells having active or inactive X appeared to be clustered within a population, suggesting that X activation status was clonal, consistent with the findings of Hanna et al [26]. Stem cells cultured in NM23-H1-MM for 14 passages were serially diluted and allowed to grow until isolated colonies were observed. The X activation status of the resultant colonies was determined by ICC staining of H3K27me3. We found that colonies that resulted from limiting dilution were either 100% XaXa or 100% XaXi. Two such clones are shown in Figure 7d and e.

**Figure 8:**
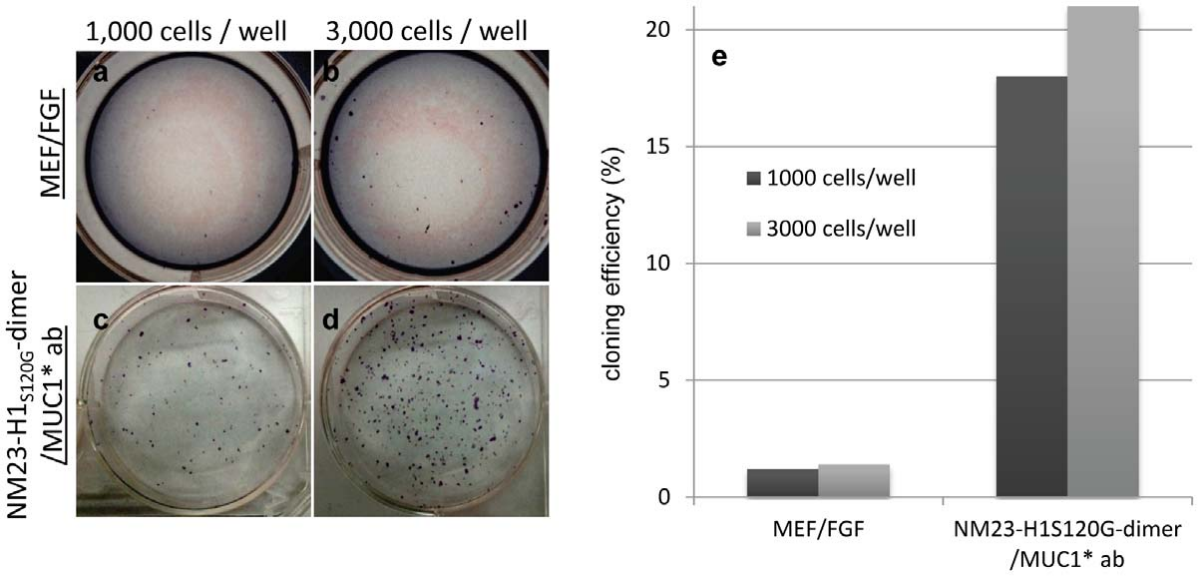
hES cells cultured in NM23-H1-MM on Anti-MUC1* surfaces have a single cell cloning efficiency that is 15 times higher than the cloning efficiency of ES cells cultures in bFGF over MEFs. a-d) Cloning efficiency of ES cells cultured in NM23-H1 media over anti-MUC1* antibody surfaces or cultured in bFGF over MEFs. Cells were plated at density of 1,000 (a and c) or 3,000 (b and d) cells/well and cultured for six days and formed colonies were stained with alkaline phosphatase. e) Stained colonies were counted and cloning efficiency was calculated. ES cells cultured in the NM23-H1 media over an anti-MUC1* antibody surface have a cloning efficiency of 21%.

Another characteristic of human stem cells in the naïve state is that they tolerate serial dissociation using trypsin, proliferate even when plated at very low densities and have increased single cell cloning efficiency compared to primed cells [26]. Trypsinization to single cells was performed routinely for all NM23-H1 cultured cells described here, but was not tolerated by bFGF-grown cells. We then compared the single cell cloning efficiency of human ES cells cultured in NM23-H1-MM over anti-MUC1* antibody surfaces to that of the same cells cultured in bFGF over MEFs. Either 1,000 (Fig. 8 a and c) or 3,000 (Fig. 8 b and d) ES cells were plated per well of a 6-well plate and allowed to grow for six days until colonies were visible. Colonies were stained for alkaline phosphatase and counted. Stem cells cultured in the NM23-H1 system reached a cloning efficiency of 21% compared to only 1.4% for cells cultured in bFGF (Fig 8e). By analogy to mouse naïve cells, we could have expected cloning efficiencies of 30% or higher. However, these experiments were performed with human stem cells, which by H3K27me3 analysis were only 50% in the pre-X-inactivation or true ground state. Yet another morphological characteristic of naïve mouse stem cells state is that they grow in dome shaped colonies rather than as flattened colonies. Human stem cells that were reverted to the naïve state by genetically modifying the cells and culturing in the presence of certain inhibitors have also reported dome shaped colony morphology. We observed that human ES and iPS cells cultured in NM23-H1-MM on antibody surfaces didn't resemble the dome shaped mouse colonies or the flattened colonies of primed cells. Cells cultured in NM23-H1-MM on antibody surfaces were flattened but grew as uniform sheets of cells rather than colonies. One reason for the morphological difference (dome versus flat) could be due to the different substrates to which the stem cells are bound. In both the genetically modified human naïve stem cells and the murine naïve cells, the substrate is MEFs, whereas we used an antibody coated surface.

## Discussion

We have demonstrated that NM23-H1-MM alone, in a defined, xeno-free media that is devoid of bFGF or any other growth factor, supports long term growth of both human ES and iPS cells on a novel defined, xeno-free surface. NM23-H1 in dimeric form binds to and dimerizes the extra cellular domain of the cleaved form of MUC1, called MUC1*. This NM23-H1-MUC1* interaction promotes pluripotency; competitive inhibition of this specific interaction induced differentiation and a concomitant spike in the expression of miR-145, a marker for exit from pluripotency. The novel surfaces we constructed are comprised of anti-MUC1* antibodies. Undifferentiated stem cells express MUC1* but revert to the quiescent full-length MUC1 as soon as the cells initiate differentiation [41]. The anti-MUC1* monoclonal antibody recognizes the clipped MUC1* form but not full-length MUC1. These MUC1* antibody surfaces not only selectively capture pluripotent stem cells but also stimulate a pathway shown to promote growth and pluripotency.

ES and iPS cells that were serially passaged in NM23-H1 media on the novel surfaces for more than 20 passages, expressed all the typical markers of pluripotency and were able to differentiate down all three germlines. Unexpectedly, stem cells grown in NM23-H1 media differentiated coordinately, often with every cell in a cluster committing to the same cell fate. This behavior was not observed for stem cells that had been cultured in media containing bFGF.

The combination of the anti-MUC1* antibody surface and our NM23-H1 media uniquely produces human stem cells that have a pattern and level of gene expression that is indicative of the stem cell naïve state. They express increased levels of markers of the “naïve” state and decreased levels of markers of the “primed” state, compared to stem cells cultured in standard bFGF-based media. ES and iPS cells cultured in this bFGF-free system also displayed other aspects that are characteristic of the naïve state. They were not adversely affected by trypsinization to single cells, could be plated at very low densities without suffering ill effects. The cloning efficiency of ES cells cultured in NM23-H1-MM over an antibody surface was 15 times greater than that of cells cultured in bFGF over MEFs. In support of the idea that MUC1* is a primal growth factor receptor that is critical to the maintenance and induction of pluripotency, we note that both OCT4 and SOX2 bind to the promoters of Muc1 and its cleavage enzyme MMP16[55].

We have demonstrated that the multimerization state of NM23-H1 determines its function. NM23-H1 dimer cooperatively binds to and dimerizes the extra cellular domain of MUC1*, which promotes growth and pluripotency; NM23-H1 hexamer does not dimerizes MUC1* and instead induces differentiation. Undifferentiated stem cells secrete NM23-H1, so as they approach some critical density when they need to initiate differentiation, the local concentration of NM23-H1 in their surrounding media consists primarily of hexamer, which triggers differentiation. Discrete multimers, like the NM23-H1 dimer and hexamer that exert opposite effects are able to exercise exquisite control over the pluripotency state and constitute an ON/OFF switch. Over a very narrow range of concentrations, NM23-H1 switches from a differentiation repressor to a differentiation inducer, making NM23-H1 the “pluripotency switch”.

## Supporting Information

Figure S1 **Protocol developed that produces recombinant NM23 as a stable population of dimer.** **a**) Recombinant NM23-wt or S120G mutants that had been purified from the soluble portionor S120G that had been denatured then refolded to form a dimer population or preparation that resulted in an approximate 50/50 mix of dimer and hexamer were analyzed on a Native gel to determine which protocols produced which multimers. Protein was loaded at 5 µg and 10 µg total protein per well. **b**) Western blot was performed on a Native gel in which the various preparations of NM23-wt or S120G mutant were loaded at very low concentrations comparable to those used in our stem cell culture (8, 16 and 32 nM). At these concentrations, NM23-wt and NM23_S120G_-hexamer were predominantly hexamer and NM23_S120G_-dimer was predominantly dimer.
(TIF)

Figure S2 **Characterization of protein expressed with the StrepTag II.** FPLC traces are shown for recombinant NM23-H1-wt, NM23-H1_S120G_-hexamer and NM23-H1_S120G_-dimer containing the Strep-tag II that were previously purified by size exclusion chromatography.
(TIF)

Figure S3 **The addition of recombinant NM23 to NM23-depleted conditioned media eliminates the need for added bFGF.** **a**) hES cells on Matrigel grew pluripotently in standard bFGF plus conditioned media from human HS27 feeder cells (control); **b**) The same cells were cultured in bFGF plus HS27 conditioned media that had been immuno-depleted of NM23 and cells immediately differentiated. **c**) Cells cultured in bFGF plus depleted conditioned media that had been reconstituted with recombinant NM23 grew pluripotently and indistinguishably from the control. **d**) Cells cultured in absence of bFGF in depleted conditioned media that had been reconstituted with recombinant NM23 grew as well as the control showing that the requirement for bFGF is eliminated by addition of recombinant NM23.
(TIF)

Figure S4 **The stability of NM23_S120G_-dimer under culture conditions was tested.** NM23_S120G_-dimer was added to cell culture media and kept in a CO2 incubator for up to 48 hours, then analyzed by western blot, which showed that no denaturation occurred within the time frame required for use in stem cell culture.
(TIF)

Figure S5 Withdrawal of growth factor NM23-H1 S120G-dimer and inhibition of NM23-H1-MUC1* interaction induce differentiation. H9 hES cells were cultured in either bFGF plus conditioned media or in NM23-H1_S120G_-dimer, and then allowed to differentiate by withholding the growth factor (a-d and e-h, respectively). Some cells also received the MUC1*_ecd_ peptide (1 µM) to competitively inhibit the NM23-H1-MUC1* interaction (i–j). Withdrawing the growth factor bFGF or in NM23-H1_S120G_-dimer induces differentiation with a maximum at 144 h. However, blocking the interaction between in NM23-H1 and MUC1* prematurely induces differentiation (96 h).
(TIF)

Figure S6 **ES and iPS cells cultured in NM23-MM grow comparably to cells cultured in bFGF as assessed by cell morphology.** **a, b**) Human H9ES cells cultured on MEFs in either NM23-MM or bFGF both appear to grow as undifferentiated stem cell colonies. **c, d**) H9s on Matrigel that were cultured in either NM23-MM or bFGF plus MEF conditioned media appear to grow comparably as pluripotent colonies. **e, f**) iPS cells cultured in NM23-MM on MEFs grew faster than the same cell line cultured in bFGF. **g**) iPS cells grew as well on Matrigel as they had on MEFs. All photos at 4X magnification.
(TIF)

Figure S7 **hES and iPS cells karyotypes.** H9s and iPS on Matrigel that had been serially passaged at least six (6) times had normal karyotype (**a and b**). H9s and iPS cells on a monoclonal anti-MUC1* antibody (MN-C3) surface that had been serially passaged at least six (6) times had normal karyotype (**c and d**).
(TIF)

Figure S8 Quantification, by fow cytometry, of the pluripotency markers expressed on the cell surface of human stem cell cultured in NM23-H1-MM over anti-MUC1* antibody surfaces. **a and d**) The pluripotency markers Tra 1-60 (a), SSEA-4 (a) and SSEA-3 (b) are expressed on the cell surface. **c**) The differenciation marker CXCR4 is barely expressed on the cell surface. d) percentage of cells expressing the different markers tested.
(TIF)

Figure S9 iPS, H14, H7 and H9 cells cultured in NM23-H1-MM on anti-MUC1* surfaces express essentially the same or higher levels of the pluripotency genes than cells cultured in bFGF on MEFs. a) A number of stem cells were cultured in either bFGF over MEFs or NM23-H1-MM over anti-MUC1* antibody MN-C3 surfaces for 10–12 passages, then assayed by RT-PCR to measure expression levels of pluripotency genes Oct4, Nanog, Klf4, and Klf2 and miR-145, an indicator of the cell's exit from pluripotency. Growth in NM23-H1-MM on anti-MUC1* ab surfaces maintains pluripotency over multiple passages for several cell lines with the same or increased expression of the pluripotency genes compared to growth in bFGF over MEFs. **b**). Welch t-test, assuming unequal variances, was used to calculate the p-values.
(TIF)

Figure S10 The difference of expression of naïve and primed markers between hES cells cultures in NM23-H1-MM over anti-MUC1* antibody surfaces and hES cells cultured in bFGF on MEFs is statistically significant. Welch t-test, assuming unequal variances, was used to calculate the p-values.
(TIF)

Figure S11 The correlation between increase of naïve marker expression and passage number of hES cells cultures in NM23-H1-MM over anti-MUC1* antibody is statistically significant. **a**) Naïve and primed gene expression scatter plot matrix for hES cells cultures in NM23-H1-MM over anti-MUC1* antibody. **b**) naïve and primed gene expression scatter plot matrix for hES cells cultures in mTeSR on Matrigel. We found a statistically significant correlation between passage number of expression in the naïve genes (r = 0.32, p<0.0001) but there was not a statistically significant correlation in the primed genes (r = −0.05, p = 0.4820) or in naïve or primed gene of cells cultures in mTeSR on Matrigel.
(TIF)

Figure S12 Expression of naïve and primed markers of human stem cells cultured over vitronectin compared to human stem cells cultured in NM23-H1-MM over anti-MUC1* antibody surfaces. H9 cells that had been serially passaged in bFGF on MEFs for 45 passages were plated onto a layer of recombinant Vitronectin and cultured in either bFGF plus MEF conditioned media, mTeSR, or NM23-H1-MM for a single passage (n = 1). All values were expressed as fold change to the control of H9 ES cells cultured in 4 ng/ml bFGF over MEFs and values for NM23-H1-MM over anti-MUC1* antibody surface is added for comparison. Overall, expression of naïve markers decreased and primed markers increased after plating onto Vitronectin.
(TIF)

Methods S1 **Supplementary Methods.**
(DOC)

## References

[1] Nelson TJ, Martinez-Fernandez A, Terzic A (2010) Induced pluripotent stem cells: developmental biology to regenerative medicine. Nat Rev Cardiol 7: 700–710. 20956984 10.1038/nrcardio.2010.159

[2] Nishikawa S-i, Goldstein RA, Nierras CR (2008) The promise of human induced pluripotent stem cells for research and therapy. Nat Rev Mol Cell Biol 9: 725–729. 18698329 10.1038/nrm2466

[3] Ludwig T, Thomson JA (2007) Defined, feeder-independent medium for human embryonic stem cell culture. Curr Protoc Stem Cell Biol Chapter 1: Unit 1C 2 10.1002/9780470151808.sc01c02s2 18785163

[4] Unger C, Skottman H, Blomberg P, Dilber MS, Hovatta O (2008) Good manufacturing practice and clinical-grade human embryonic stem cell lines. Hum Mol Genet 17: R48–53. 18632697 10.1093/hmg/ddn079

[5] Akopian V, Andrews PW, Beil S, Benvenisty N, Brehm J, et al (2010) Comparison of defined culture systems for feeder cell free propagation of human embryonic stem cells. In Vitro Cell Dev Biol Anim 46: 247–258. 20186512 10.1007/s11626-010-9297-z PMC2855804

[6] Klimanskaya I, Chung Y, Meisner L, Johnson J, West MD, et al (2005) Human embryonic stem cells derived without feeder cells. Lancet 365: 1636–1641. 15885296 10.1016/S0140-6736(05)66473-2

[7] Richards M, Fong C-Y, Chan W-K, Wong P-C, Bongso A (2002) Human feeders support prolonged undifferentiated growth of human inner cell masses and embryonic stem cells. Nat Biotech 20: 933–936. 10.1038/nbt726 12161760

[8] Thomson JA, Itskovitz-Eldor J, Shapiro SS, Waknitz MA, Swiergiel JJ, et al (1998) Embryonic Stem Cell Lines Derived from Human Blastocysts. Science 282: 1145–1147. 9804556 10.1126/science.282.5391.1145

[9] Xu C, Inokuma MS, Denham J, Golds K, Kundu P, et al (2001) Feeder-free growth of undifferentiated human embryonic stem cells. Nat Biotech 19: 971–974. 10.1038/nbt1001-971 11581665

[10] Kleinman HK, Martin GR (2005) Matrigel: Basement membrane matrix with biological activity. Seminars in Cancer Biology 15: 378–386. 15975825 10.1016/j.semcancer.2005.05.004

[11] Orkin RW, Gehron P, McGoodwin EB, Martin GR, Valentine T, et al (1977) A,urine tumor producing a matrix of basement membrane. The journal of experimental medicine 145: 204–220. 830788 10.1084/jem.145.1.204 PMC2180589

[12] Chen G, Gulbranson DR, Hou Z, Bolin JM, Ruotti V, et al (2011) Chemically defined conditions for human iPSC derivation and culture. Nat Methods 8: 424–429. 21478862 10.1038/nmeth.1593 PMC3084903

[13] Pompe T, Salchert K, Alberti K, Zandstra P, Werner C (2010) Immobilization of growth factors on solid supports for the modulation of stem cell fate. Nat Protocols 5: 1042–1050. 20539280 10.1038/nprot.2010.70

[14] Yang MT, Fu J, Wang Y-K, Desai RA, Chen CS (2011) Assaying stem cell mechanobiology on microfabricated elastomeric substrates with geometrically modulated rigidity. Nat Protocols 6: 187–213. 21293460 10.1038/nprot.2010.189 PMC7183577

[15] Braam SR, Zeinstra L, Litjens S, Ward-van Oostwaard D, van den Brink S, et al (2008) Recombinant Vitronectin Is a Functionally Defined Substrate That Supports Human Embryonic Stem Cell Self-Renewal via αVβ5 Integrin. STEM CELLS 26: 2257–2265. 18599809 10.1634/stemcells.2008-0291

[16] Derda R, Musah S, Orner BP, Klim JR, Li L, et al (2010) High-Throughput Discovery of Synthetic Surfaces That Support Proliferation of Pluripotent Cells. Journal of the American Chemical Society 132: 1289–1295. 20067240 10.1021/ja906089g PMC2819098

[17] Klim JR, Fowler AJ, Courtney AH, Wrighton PJ, Sheridan RTC, et al (2012) Small-Molecule-Modified Surfaces Engage Cells through the Î±vÎ^2^3 Integrin. ACS Chemical Biology 10.1021/cb2004725 PMC3306508 22201290

[18] Klim JR, Li L, Wrighton PJ, Piekarczyk MS, Kiessling LL (2010) A defined glycosaminoglycan-binding substratum for human pluripotent stem cells. Nat Meth 7: 989–994. 10.1038/nmeth.1532 PMC2994976 21076418

[19] Li YJ, Chung EH, Rodriguez RT, Firpo MT, Healy KE (2006) Hydrogels as artificial matrices for human embryonic stem cell self-renewal. Journal of Biomedical Materials Research Part A 79A: 1–5. 10.1002/jbm.a.30732 16741988

[20] Melkoumian Z, Weber JL, Weber DM, Fadeev AG, Zhou Y, et al (2010) Synthetic peptide-acrylate surfaces for long-term self-renewal and cardiomyocyte differentiation of human embryonic stem cells. Nat Biotech 28: 606–610. 10.1038/nbt.1629 20512120

[21] Meng Y, Eshghi S, Li YJ, Schmidt R, Schaffer DV, et al (2010) Characterization of integrin engagement during defined human embryonic stem cell culture. The journal of the Federation of American Societies for Experimental Biology 24: 1056–1065. 10.1096/fj.08-126821 PMC2845424 19933311

[22] Saha K, Mei Y, Reisterer CM, Pyzocha NK, Yang J, et al (2011) Surface-engineered substrates for improved human pluripotent stem cell culture under fully defined conditions. Proc Natl Acad Sci U S A 108: 18714–18719. 22065768 10.1073/pnas.1114854108 PMC3219112

[23] Villa-Diaz LG, Nandivada H, Ding J, Nogueira-de-Souza NC, Krebsbach PH, et al (2010) Synthetic polymer coatings for long-term growth of human embryonic stem cells. Nat Biotech 28: 581–583. 10.1038/nbt.1631 PMC3471651 20512122

[24] Vuoristo S, Virtanen I, Takkunen M, Palgi J, Kikkawa Y, et al (2009) Laminin isoforms in human embryonic stem cells: synthesis, receptor usage and growth support. Journal of Cellular and Molecular Medicine 13: 2622–2633. 19397785 10.1111/j.1582-4934.2008.00643.x PMC6529980

[25] Nichols J, Smith A (2009) Naive and primed pluripotent states. Cell Stem Cell 4: 487–492. 19497275 10.1016/j.stem.2009.05.015

[26] Hanna J, Cheng AW, Saha K, Kim J, Lengner CJ, et al (2010) Human embryonic stem cells with biological and epigenetic characteristics similar to those of mouse ESCs. Proc Natl Acad Sci U S A 107: 9222–9227. 20442331 10.1073/pnas.1004584107 PMC2889088

[27] Amit M, Carpenter MK, Inokuma MS, Chiu C-P, Harris CP, et al (2000) Clonally Derived Human Embryonic Stem Cell Lines Maintain Pluripotency and Proliferative Potential for Prolonged Periods of Culture. Developmental Biology 227: 271–278. 11071754 10.1006/dbio.2000.9912

[28] Ludwig TE, Levenstein ME, Jones JM, Berggren WT, Mitchen ER, et al (2006) Derivation of human embryonic stem cells in defined conditions. Nat Biotechnol 24: 185–187. 16388305 10.1038/nbt1177

[29] Xu C, Rosler E, Jiang J, Lebkowski JS, Gold JD, et al (2005) Basic Fibroblast Growth Factor Supports Undifferentiated Human Embryonic Stem Cell Growth Without Conditioned Medium. STEM CELLS 23: 315–323. 15749926 10.1634/stemcells.2004-0211

[30] Xu RH, Peck RM, Li DS, Feng X, Ludwig T, et al (2005) Basic FGF and suppression of BMP signaling sustain undifferentiated proliferation of human ES cells. Nat Methods 2: 185–190. 15782187 10.1038/nmeth744

[31] Steeg PS, Bevilacqua G, Kopper L, Thorgeirsson UP, Talmadge JE, et al (1988) Evidence for a Novel Gene Associated With Low Tumor Metastatic Potential. J Natl Cancer Inst 80: 200–204. 3346912 10.1093/jnci/80.3.200

[32] Hozumi M (1985) Established leukemia cell lines: their role in the understanding and control of leukemia proliferation. Crit Rev Oncol Hematol 3: 235–277. 3899390 10.1016/s1040-8428(85)80028-7

[33] Okabe-Kado J, Kasukabe T, Honma Y, Hayashi M, Henzel WJ, et al (1992) Identity of a differentiation inhibiting factor for mouse myeloid leukemia cells with NM23/nucleoside diphosphate kinase. Biochemical and Biophysical Research Communications 182: 987–994. 1311576 10.1016/0006-291x(92)91829-f

[34] Biggs J, Hersperger E, Steeg PS, Liotta LA, Shearn A (1990) A Drosophila gene that is homologous to a mammalian gene associated with tumor metastasis codes for a nucleoside diphosphate kinase. Cell 63: 933–940. 2175255 10.1016/0092-8674(90)90496-2

[35] Wallet Vr, Mutzel R, Troll H, Barzu O, Wurster B, et al (1990) Dictyostelium Nucleoside Diphospate Kinase Highly Homologous to Nm23 and Awd Proteins Involved in Mammalian Tumor Metastasis and Drosphila Development. pp 1199–1202. 10.1093/jnci/82.14.1199 2163458

[36] Okabe-Kado J, Kasukabe T, Hozumi M, Honma Y, Kimura N, et al (1995) A new function of Nm23/NDP kinase as a differentiation inhibitory factor, which does not require it's kinase activity. FEBS Letters 363: 311–315. 7737424 10.1016/0014-5793(95)00338-a

[37] Lombardi D, Lacombe M-L, Paggi MG (2000) nm23: Unraveling its biological function in cell differentiation. Journal of Cellular Physiology 182: 144–149. 10623877 10.1002/(SICI)1097-4652(200002)182:2<144::AID-JCP2>3.0.CO;2-6

[38] Mesnildrey Sb, Agou F, Karlsson A, Bonne DD, VÃ©ron M (1998) Coupling between Catalysis and Oligomeric Structure in Nucleoside Diphosphate Kinase. pp 4436–4442. 10.1074/jbc.273.8.4436 9468495

[39] Mesnildrey Sb, Agou F, VÃ©ron M (1997) The in vitro DNA binding properties of NDP kinase are related to its oligomeric state. FEBS Letters 418: 53–57. 9414094 10.1016/s0014-5793(97)01292-1

[40] Song EJ, Kim YS, Chung JY, Kim E, Chae S-K, et al (2000) Oxidative Modification of Nucleoside Diphosphate Kinase and Its Identification by Matrix-Assisted Laser Desorption/Ionization Time-of-Flight Mass Spectrometryâ Biochemistry. 39: 10090–10097. 10.1021/bi000267a 10955997

[41] Hikita ST, Kosik KS, Clegg DO, Bamdad C (2008) MUC1* mediates the growth of human pluripotent stem cells. PLoS One 3: e3312. 18833326 10.1371/journal.pone.0003312 PMC2553196

[42] Mahanta S, Fessler SP, Park J, Bamdad C (2008) A minimal fragment of MUC1 mediates growth of cancer cells. PLoS One 3: e2054. 18446242 10.1371/journal.pone.0002054 PMC2329594

[43] Bamdad C (1998) The Use of Variable Density Self-Assembled Monolayers to Probe the Structure of a Target Molecule. Biophysical Journal 75: 1989–1996. 9746540 10.1016/S0006-3495(98)77640-4 PMC1299870

[44] Thompson AB, Calhoun AK, Smagghe BJ, Stevens MD, Wotkowicz MT, et al (2011) A Gold Nanoparticle Platform for Proteinâ€“Protein Interactions and Drug Discovery. ACS Applied Materials & Interfaces. 3: 2979–2987. 10.1021/am200459a 21699220

[45] Kim Y-I, Park S, Jeoung D-I, Lee H (2003) Point mutations affecting the oligomeric structure of Nm23-H1 abrogates its inhibitory activity on colonization and invasion of prostate cancer cells. Biochemical and Biophysical Research Communications 307: 281–289. 12859952 10.1016/s0006-291x(03)01195-1

[46] Lascu I, Schaertl S, Wang C, Sarger C, Giartosio A, et al (1997) A Point Mutation of Human Nucleoside Diphosphate Kinase A Found in Aggressive Neuroblastoma Affects Protein Folding. J Biol Chem 272: 15599–15602. 9188446 10.1074/jbc.272.25.15599

[47] Chang CL, Zhu X-x, Thoraval DH, Ungar D, Rawwas J, et al (1994) nm23-H1 mutation in neuroblastoma. Nature 370: 335–336. 8047138 10.1038/370335a0

[48] Liedberg B, Nylander C, LunstrÃ¶m I (1983) Surface plasmon resonance for gas detection and biosensing. Sensors and Actuators 4: 299–304.

[49] Bamdad C, Bamdad RS (1999) Rapid and sensitive detection of aberrant protein aggregation in neurodegenerative disease.

[50] Liedberg B, Lundstrom I, Stenberg E (1993) Principles of biosensing with an extended coupling matrix and surface plasmon resonance. Sensors and Actuators B: Chemical 11: 63–72.

[51] Xu N, Papagiannakopoulos T, Pan G, Thomson JA, Kosik KS (2009) MicroRNA-145 regulates OCT4, SOX2, and KLF4 and represses pluripotency in human embryonic stem cells. Cell 137: 647–658. 19409607 10.1016/j.cell.2009.02.038

[52] Doetschman TC, Eistetter H, Katz M, Schmidt W, Kemler R (1985) The in vitro development of blastocyst-derived embryonic stem cell lines: formation of visceral yolk sac, blood islands and myocardium. J Embryol Exp Morphol 87: 27–45. 3897439

[53] Hiroshi K (2007) Methods for inducing embryoid body formation: in vitro differentiation system of embryonic stem cells. Journal of Bioscience and Bioengineering 103: 389–398. 17609152 10.1263/jbb.103.389

[54] Watanabe K, Ueno M, Kamiya D, Nishiyama A, Matsumura M, et al (2007) A ROCK inhibitor permits survival of dissociated human embryonic stem cells. Nat Biotech 25: 681–686. 10.1038/nbt1310 17529971

[55] Boyer LA, Lee TI, Cole MF, Johnstone SE, Levine SS, et al (2005) Core Transcriptional Regulatory Circuitry in Human Embryonic Stem Cells. Cell 122: 947–956. 16153702 10.1016/j.cell.2005.08.020 PMC3006442

