# Supplementary figures and images for: MUC1* Ligand, NM23-H1, Is a Novel Growth Factor That Maintains Human Stem Cells in a More Naïve State
Source: PLoS One. 2013 Mar 7;8(3):e58601. doi: 10.1371/journal.pone.0058601 (PMC3591366; doi:10.1371/journal.pone.0058601)

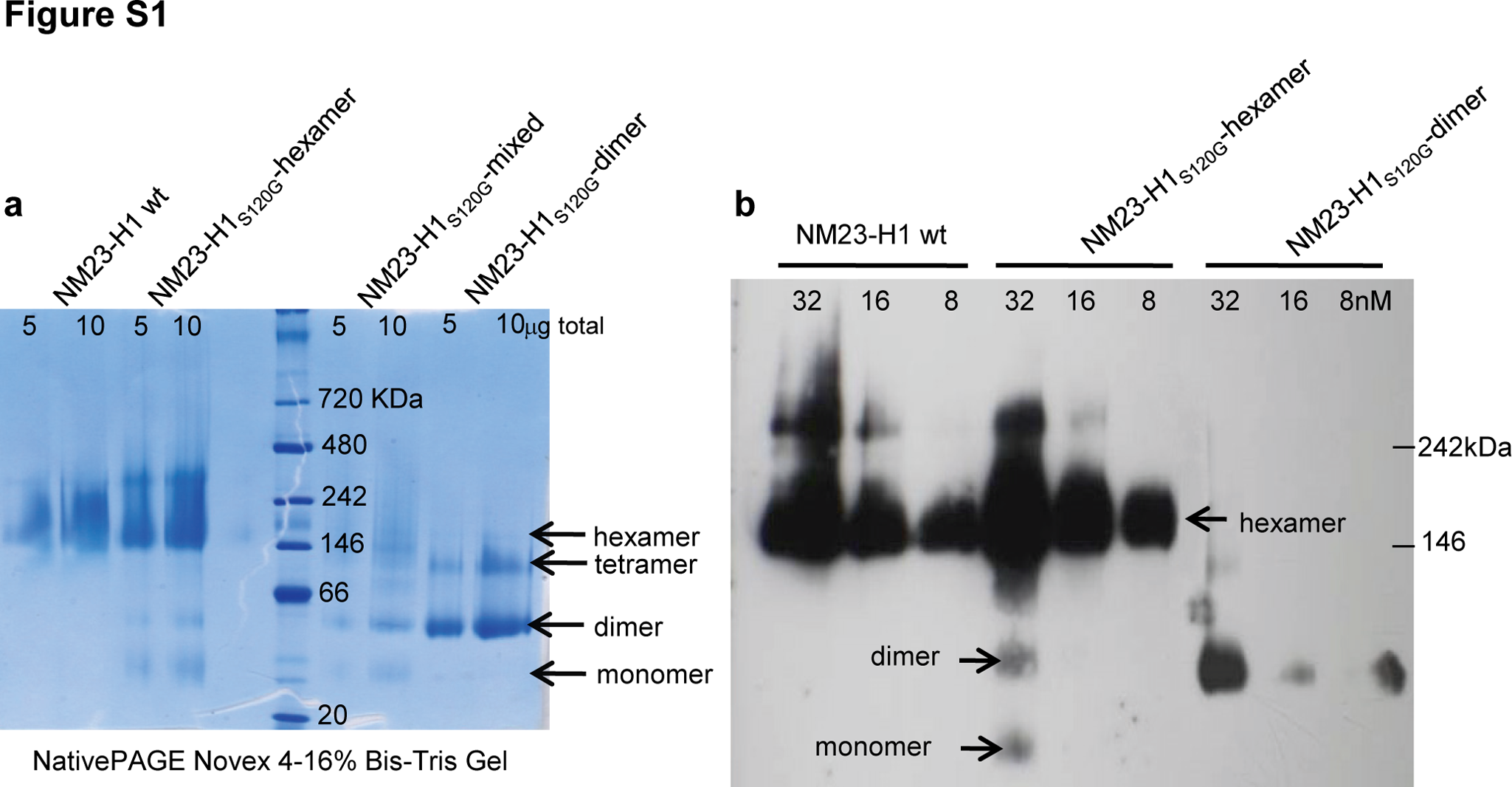

Supplement: Figure S1 — Protocol developed that produces recombinant NM23 as a stable population of dimer. a) Recombinant NM23-wt or S120G mutants that had been purified from the soluble portionor S120G that had been denatured then refolded to form a dimer population or preparation that resulted in an approximate 50/50 mix of dimer and hexamer were analyzed on a Native gel to determine which protocols produced which multimers. Protein was loaded at 5 µg and 10 µg total protein per well. b) Western blot was performed on a Native gel in which the various preparations of NM23-wt or S120G mutant were loaded at very low concentrations comparable to those used in our stem cell culture (8, 16 and 32 nM). At these concentrations, NM23-wt and NM23S120G-hexamer were predominantly hexamer and NM23S120G-dimer was predominantly dimer. (TIF) [file pone.0058601.s001.tif]

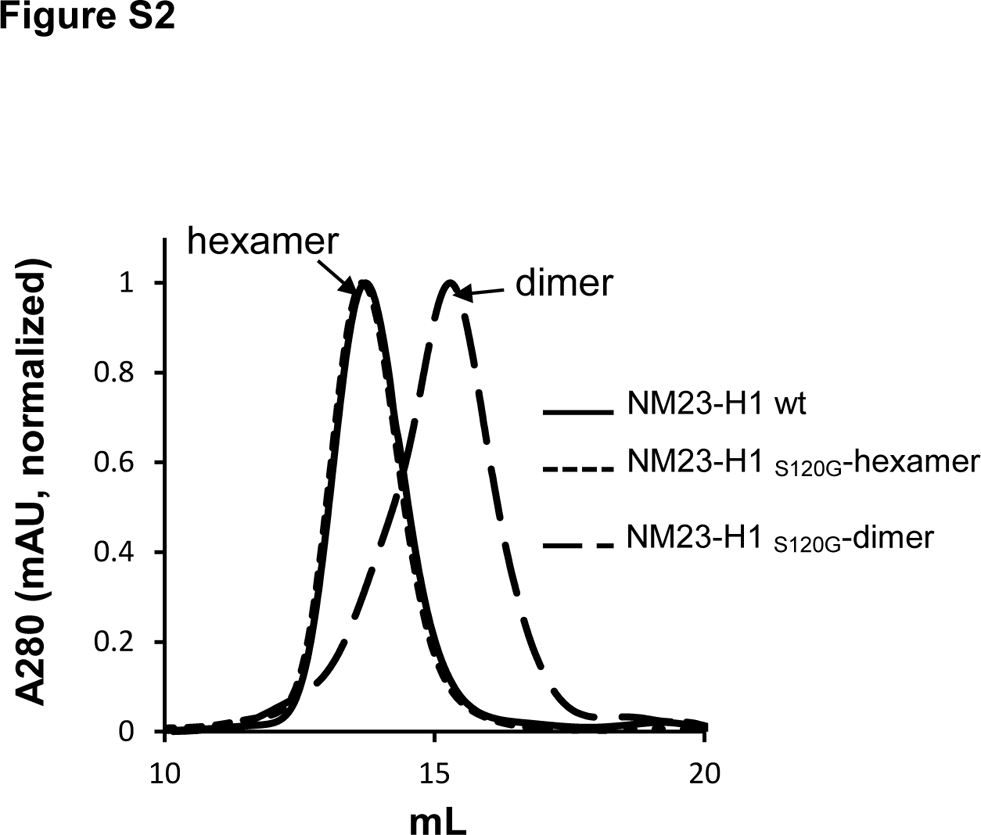

Supplement: Figure S2 — Characterization of protein expressed with the StrepTag II. FPLC traces are shown for recombinant NM23-H1-wt, NM23-H1S120G-hexamer and NM23-H1S120G-dimer containing the Strep-tag II that were previously purified by size exclusion chromatography. (TIF) [file pone.0058601.s002.tif]

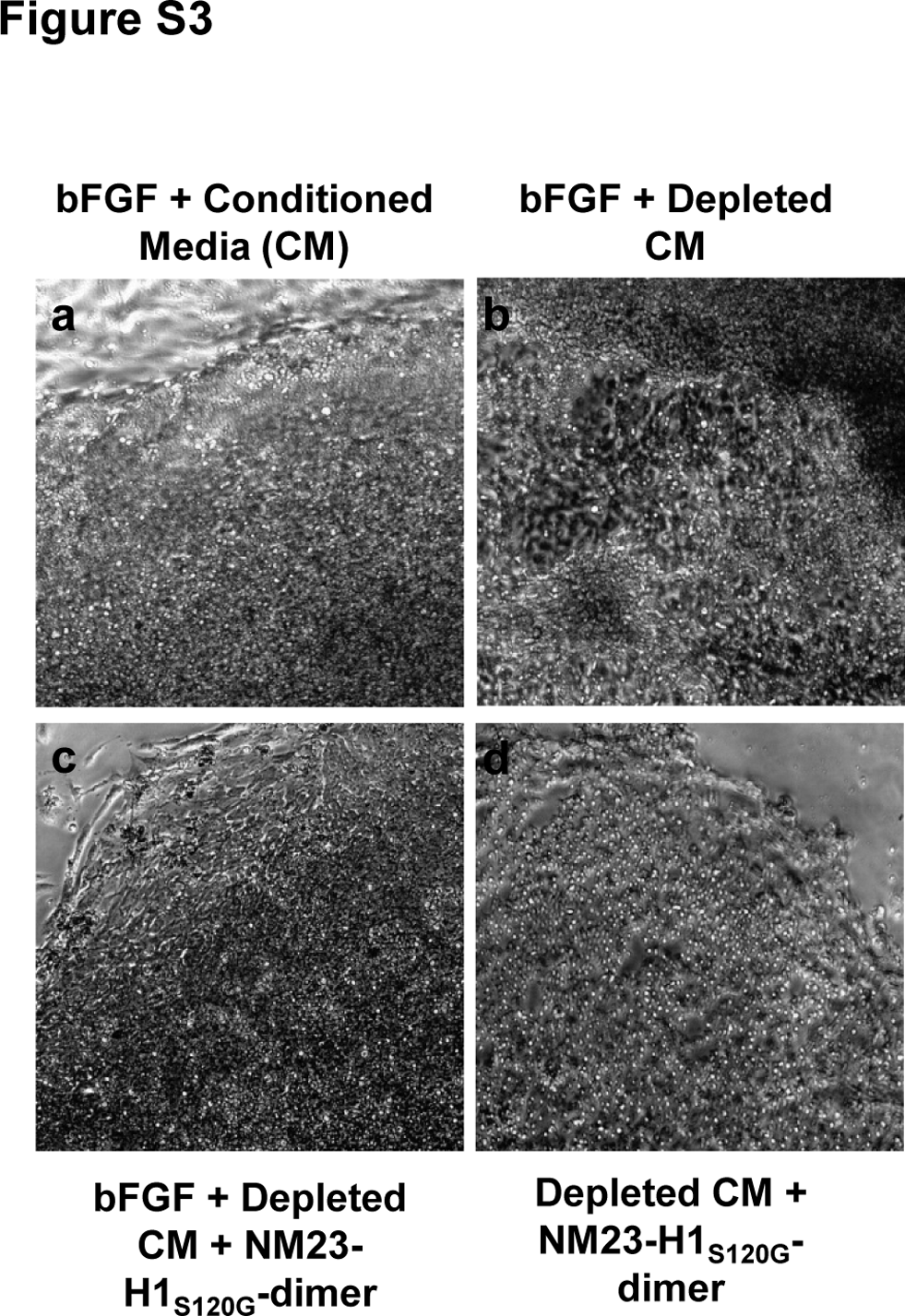

Supplement: Figure S3 — The addition of recombinant NM23 to NM23-depleted conditioned media eliminates the need for added bFGF. a) hES cells on Matrigel grew pluripotently in standard bFGF plus conditioned media from human HS27 feeder cells (control); b) The same cells were cultured in bFGF plus HS27 conditioned media that had been immuno-depleted of NM23 and cells immediately differentiated. c) Cells cultured in bFGF plus depleted conditioned media that had been reconstituted with recombinant NM23 grew pluripotently and indistinguishably from the control. d) Cells cultured in absence of bFGF in depleted conditioned media that had been reconstituted with recombinant NM23 grew as well as the control showing that the requirement for bFGF is eliminated by addition of recombinant NM23. (TIF) [file pone.0058601.s003.tif]

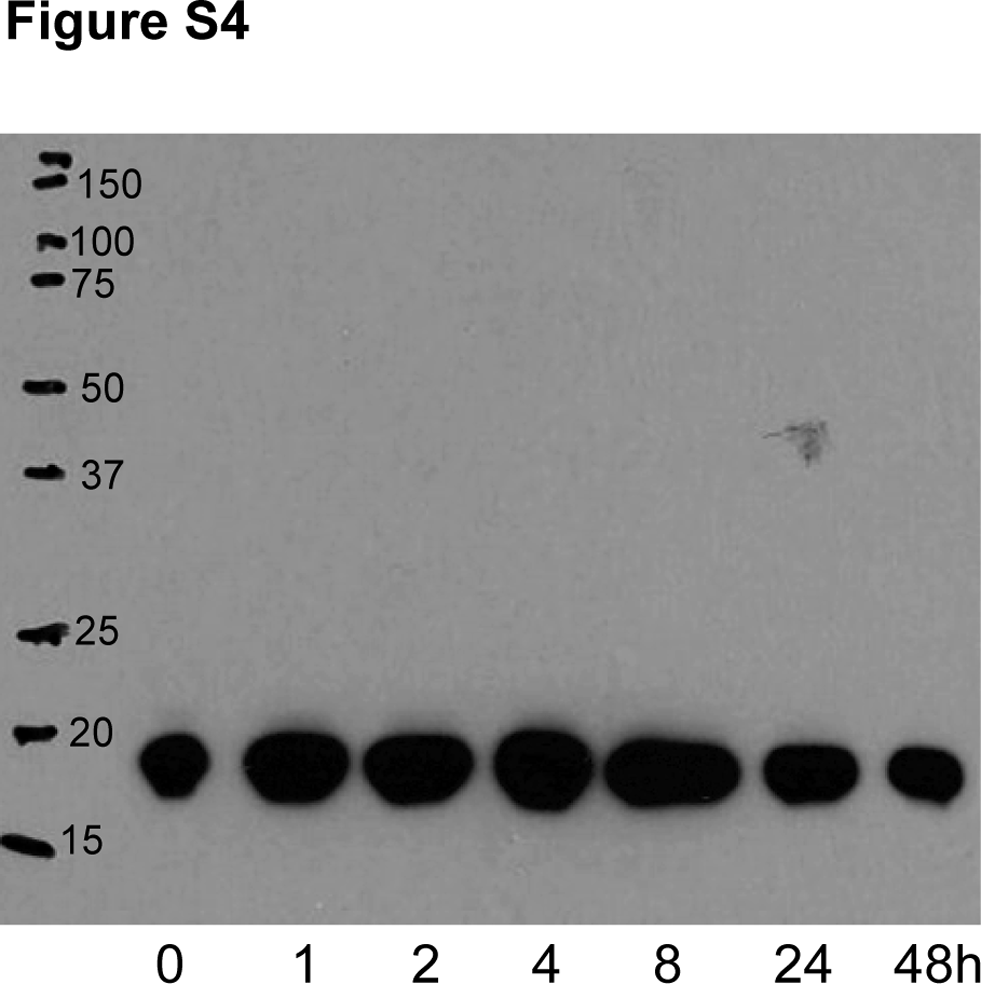

Supplement: Figure S4 — The stability of NM23S120G-dimer under culture conditions was tested. NM23S120G-dimer was added to cell culture media and kept in a CO2 incubator for up to 48 hours, then analyzed by western blot, which showed that no denaturation occurred within the time frame required for use in stem cell culture. (TIF) [file pone.0058601.s004.tif]

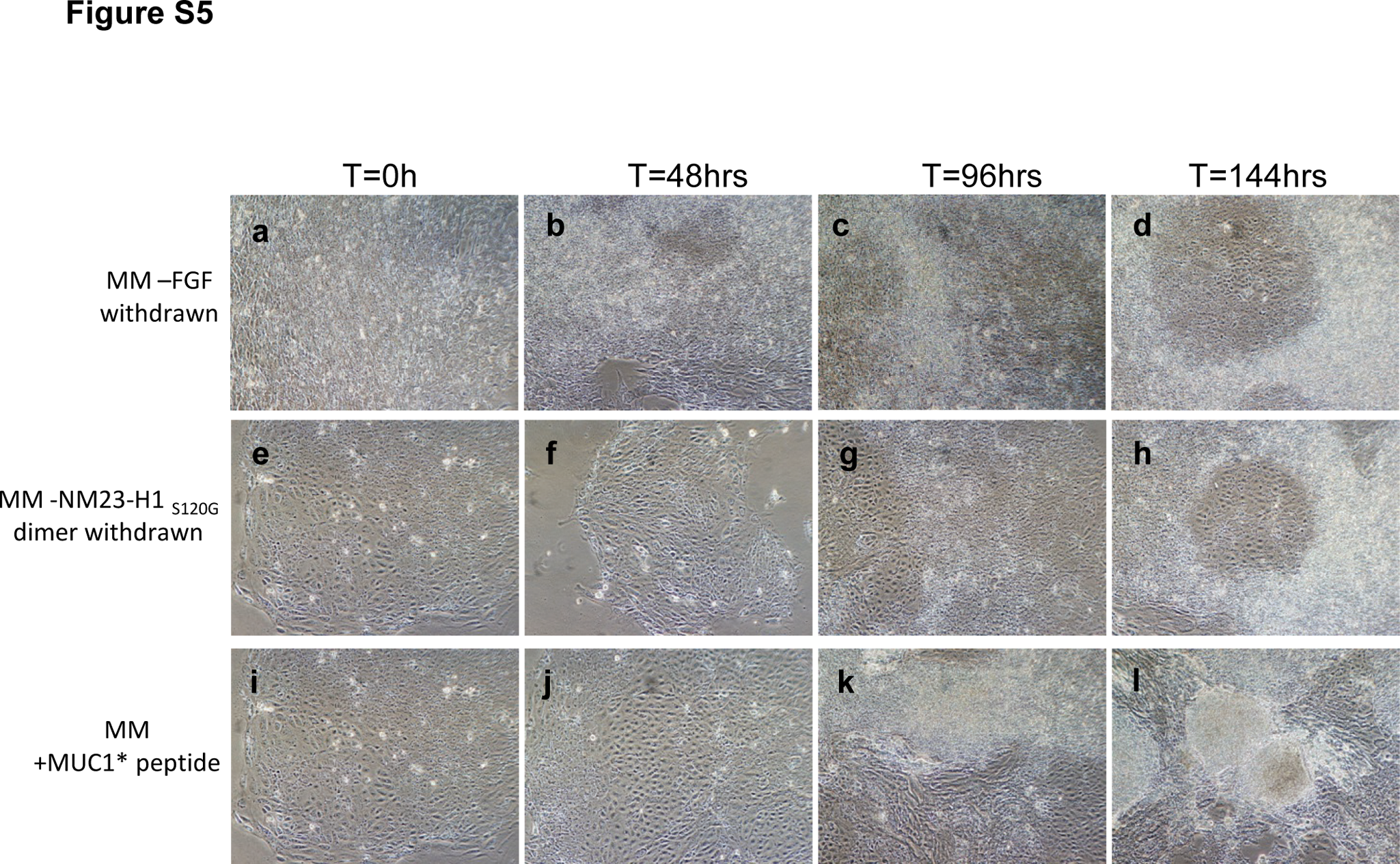

Supplement: Figure S5 — Withdrawal of growth factor NM23-H1 S120G-dimer and inhibition of NM23-H1-MUC1* interaction induce differentiation. H9 hES cells were cultured in either bFGF plus conditioned media or in NM23-H1S120G-dimer, and then allowed to differentiate by withholding the growth factor (a-d and e-h, respectively). Some cells also received the MUC1*ecd peptide (1 µM) to competitively inhibit the NM23-H1-MUC1* interaction (i–j). Withdrawing the growth factor bFGF or in NM23-H1S120G-dimer induces differentiation with a maximum at 144 h. However, blocking the interaction between in NM23-H1 and MUC1* prematurely induces differentiation (96 h). (TIF) [file pone.0058601.s005.tif]

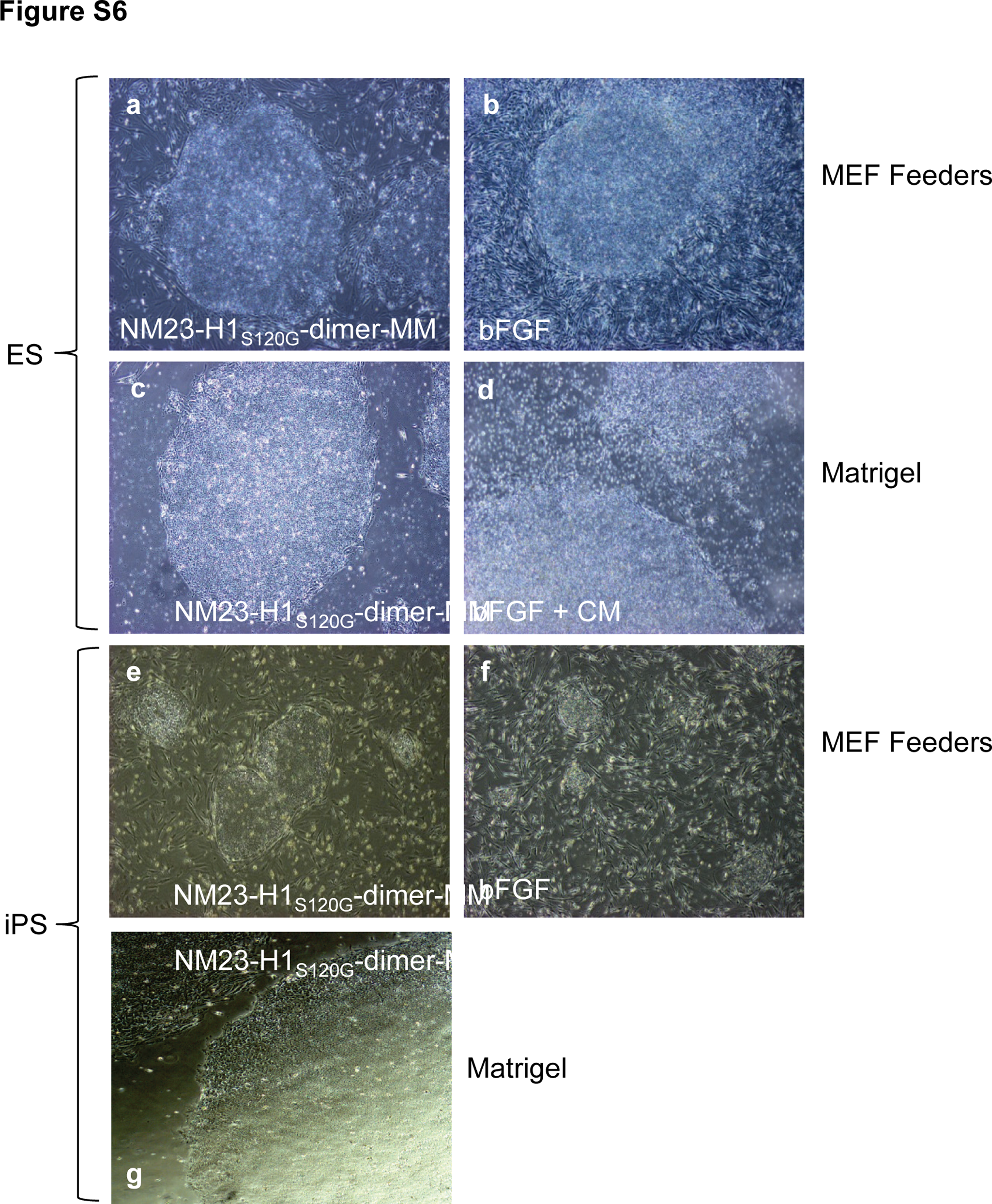

Supplement: Figure S6 — ES and iPS cells cultured in NM23-MM grow comparably to cells cultured in bFGF as assessed by cell morphology. a, b) Human H9ES cells cultured on MEFs in either NM23-MM or bFGF both appear to grow as undifferentiated stem cell colonies. c, d) H9s on Matrigel that were cultured in either NM23-MM or bFGF plus MEF conditioned media appear to grow comparably as pluripotent colonies. e, f) iPS cells cultured in NM23-MM on MEFs grew faster than the same cell line cultured in bFGF. g) iPS cells grew as well on Matrigel as they had on MEFs. All photos at 4X magnification. (TIF) [file pone.0058601.s006.tif]

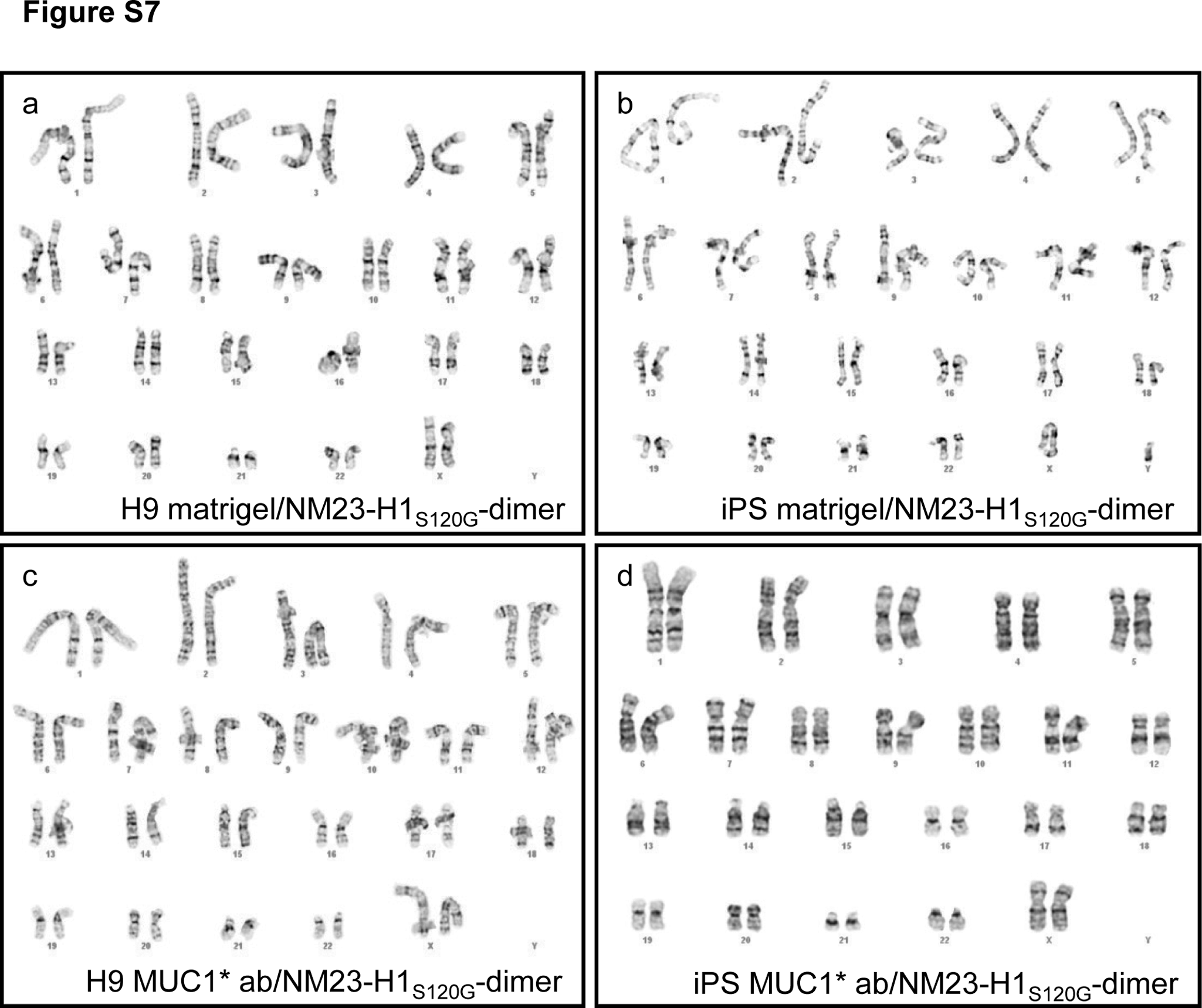

Supplement: Figure S7 — hES and iPS cells karyotypes. H9s and iPS on Matrigel that had been serially passaged at least six (6) times had normal karyotype (a and b). H9s and iPS cells on a monoclonal anti-MUC1* antibody (MN-C3) surface that had been serially passaged at least six (6) times had normal karyotype (c and d). (TIF) [file pone.0058601.s007.tif]

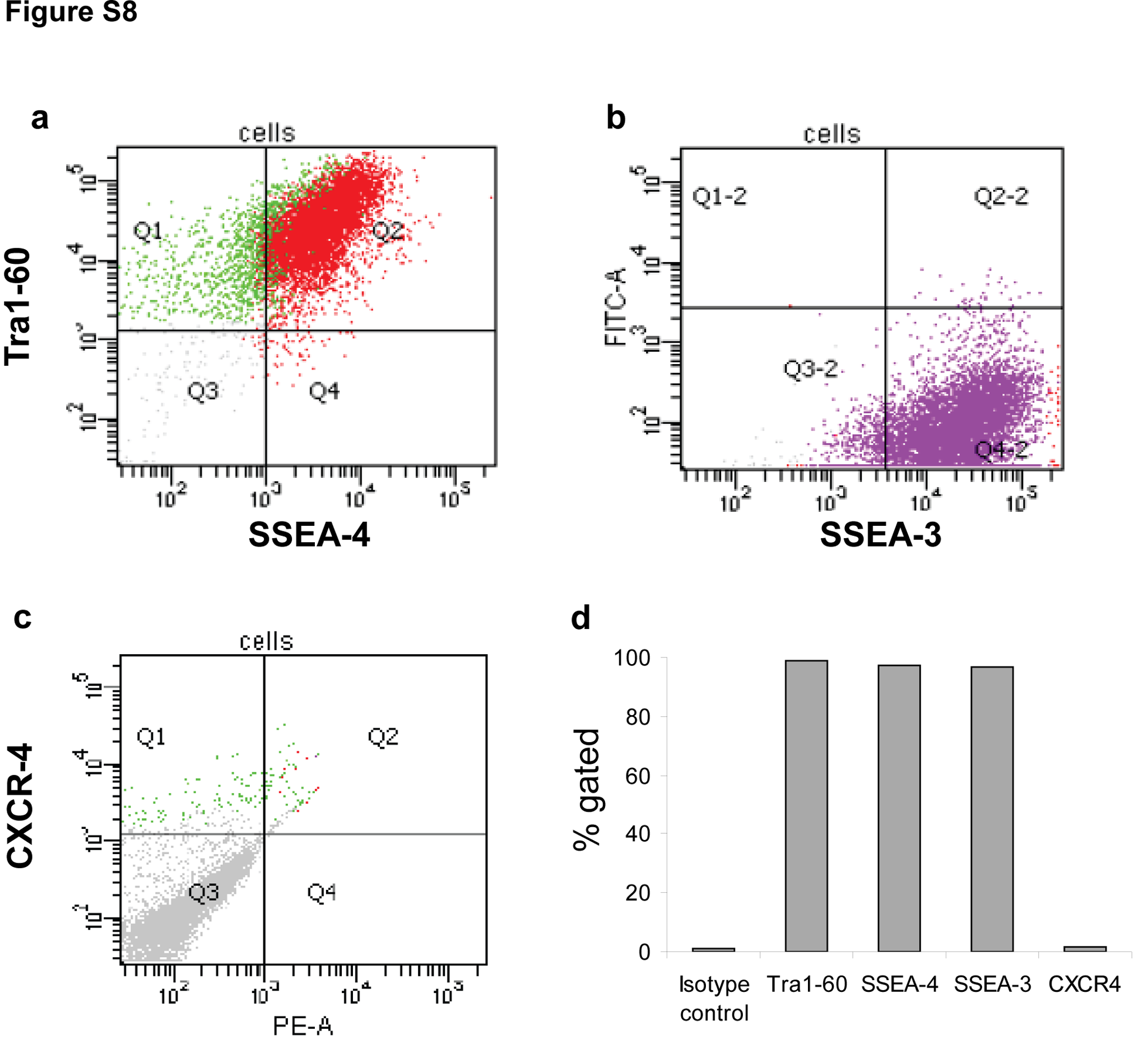

Supplement: Figure S8 — Quantification, by fow cytometry, of the pluripotency markers expressed on the cell surface of human stem cell cultured in NM23-H1-MM over anti-MUC1* antibody surfaces. a and d) The pluripotency markers Tra 1-60 (a), SSEA-4 (a) and SSEA-3 (b) are expressed on the cell surface. c) The differenciation marker CXCR4 is barely expressed on the cell surface. d) percentage of cells expressing the different markers tested. (TIF) [file pone.0058601.s008.tif]

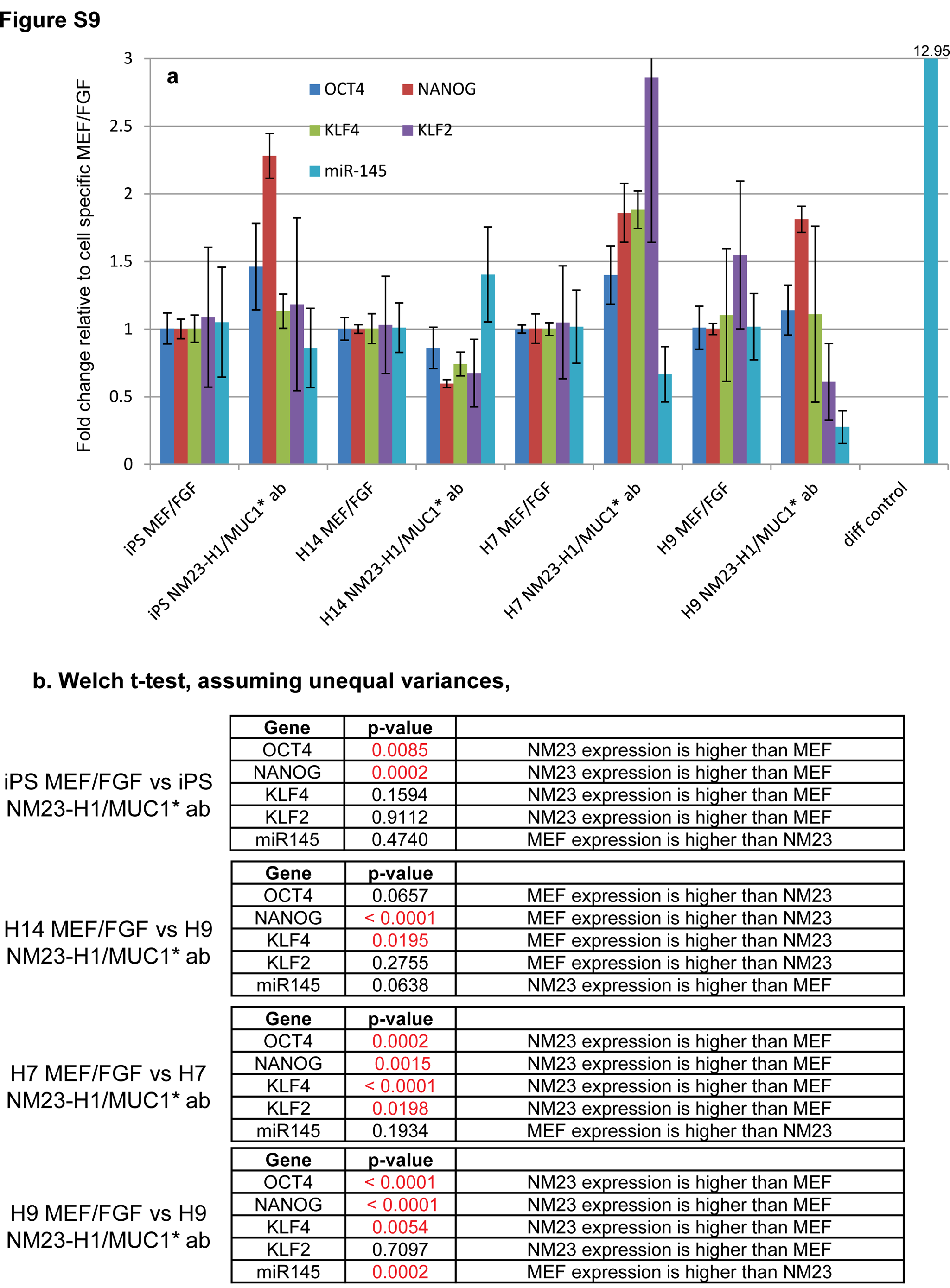

Supplement: Figure S9 — iPS, H14, H7 and H9 cells cultured in NM23-H1-MM on anti-MUC1* surfaces express essentially the same or higher levels of the pluripotency genes than cells cultured in bFGF on MEFs. a) A number of stem cells were cultured in either bFGF over MEFs or NM23-H1-MM over anti-MUC1* antibody MN-C3 surfaces for 10–12 passages, then assayed by RT-PCR to measure expression levels of pluripotency genes Oct4, Nanog, Klf4, and Klf2 and miR-145, an indicator of the cell's exit from pluripotency. Growth in NM23-H1-MM on anti-MUC1* ab surfaces maintains pluripotency over multiple passages for several cell lines with the same or increased expression of the pluripotency genes compared to growth in bFGF over MEFs. b). Welch t-test, assuming unequal variances, was used to calculate the p-values. (TIF) [file pone.0058601.s009.tif]

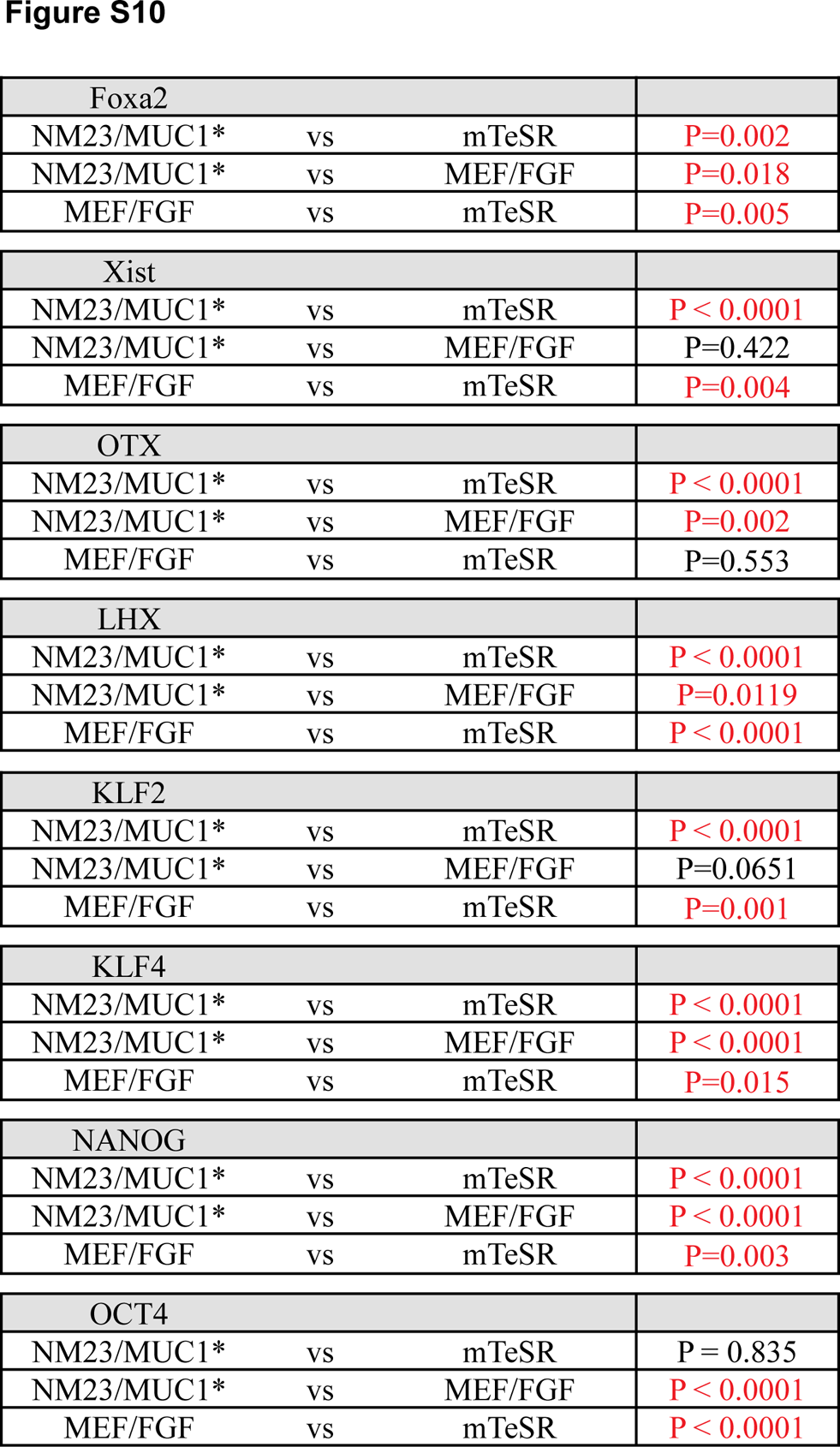

Supplement: Figure S10 — The difference of expression of naïve and primed markers between hES cells cultures in NM23-H1-MM over anti-MUC1* antibody surfaces and hES cells cultured in bFGF on MEFs is statistically significant. Welch t-test, assuming unequal variances, was used to calculate the p-values. (TIF) [file pone.0058601.s010.tif]

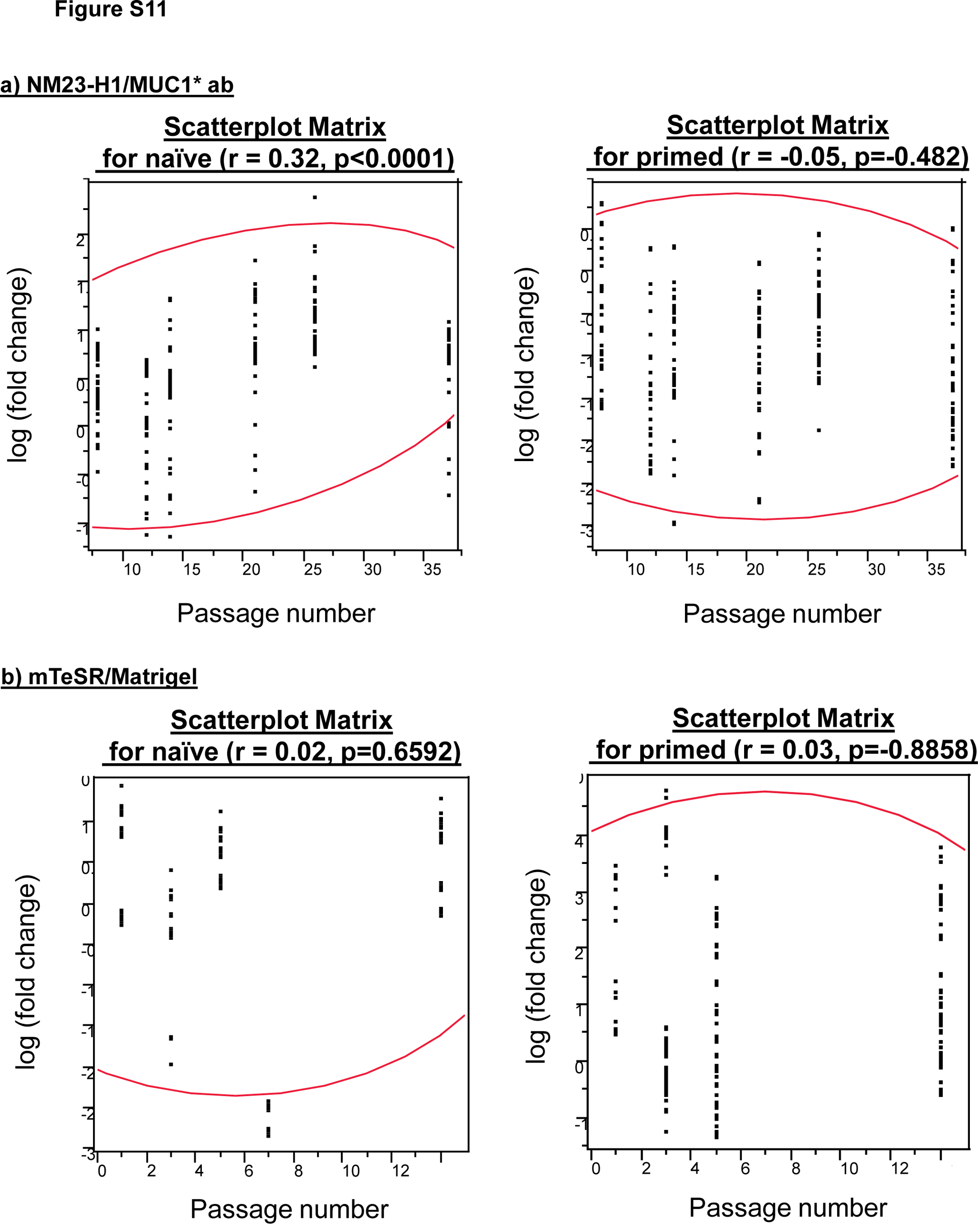

Supplement: Figure S11 — The correlation between increase of naïve marker expression and passage number of hES cells cultures in NM23-H1-MM over anti-MUC1* antibody is statistically significant. a) Naïve and primed gene expression scatter plot matrix for hES cells cultures in NM23-H1-MM over anti-MUC1* antibody. b) naïve and primed gene expression scatter plot matrix for hES cells cultures in mTeSR on Matrigel. We found a statistically significant correlation between passage number of expression in the naïve genes (r = 0.32, p<0.0001) but there was not a statistically significant correlation in the primed genes (r = −0.05, p = 0.4820) or in naïve or primed gene of cells cultures in mTeSR on Matrigel. (TIF) [file pone.0058601.s011.tif]

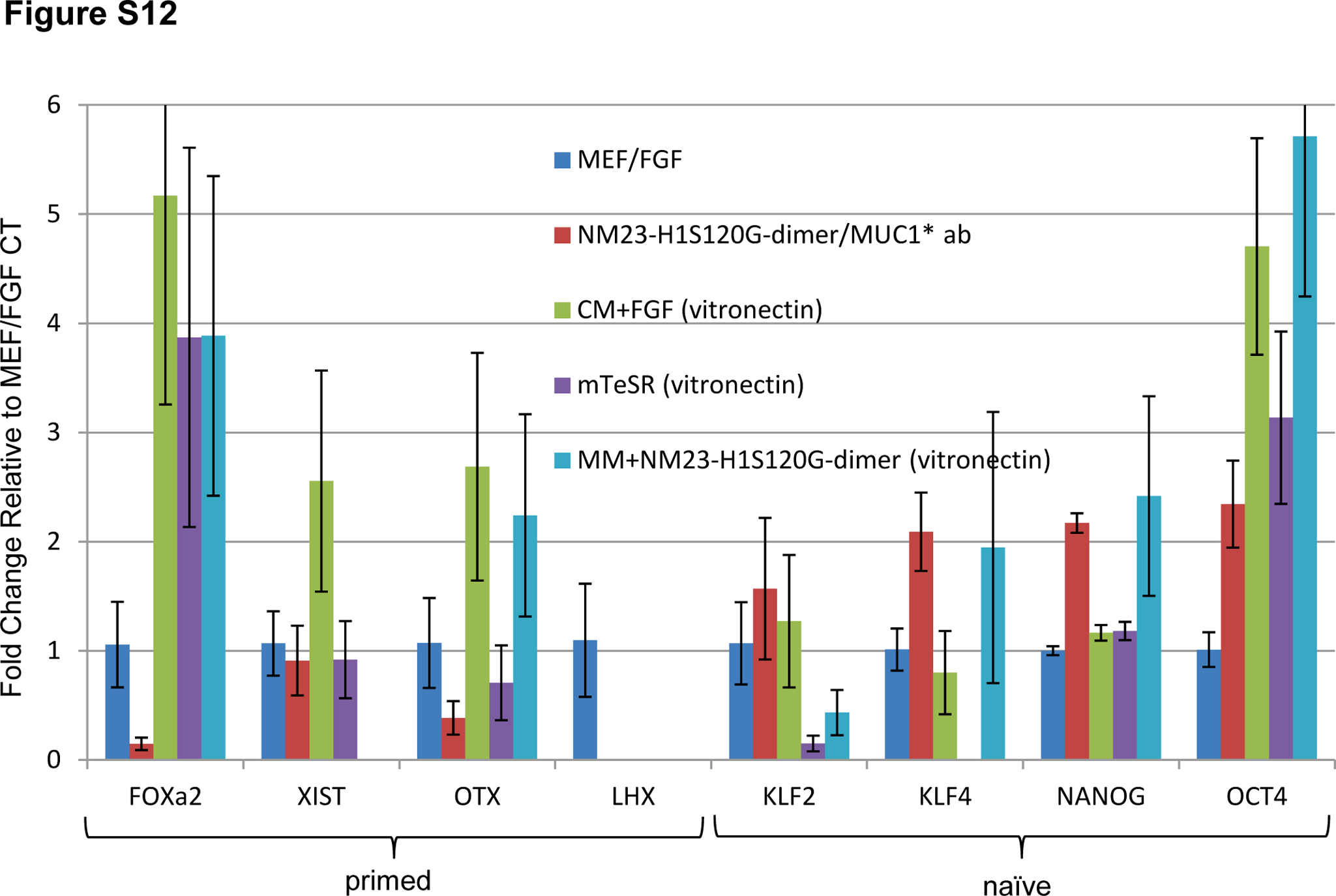

Supplement: Figure S12 — Expression of naïve and primed markers of human stem cells cultured over vitronectin compared to human stem cells cultured in NM23-H1-MM over anti-MUC1* antibody surfaces. H9 cells that had been serially passaged in bFGF on MEFs for 45 passages were plated onto a layer of recombinant Vitronectin and cultured in either bFGF plus MEF conditioned media, mTeSR, or NM23-H1-MM for a single passage (n = 1). All values were expressed as fold change to the control of H9 ES cells cultured in 4 ng/ml bFGF over MEFs and values for NM23-H1-MM over anti-MUC1* antibody surface is added for comparison. Overall, expression of naïve markers decreased and primed markers increased after plating onto Vitronectin. (TIF) [file pone.0058601.s012.tif]
