# Supplementary material for: MUC1* Ligand, NM23-H1, Is a Novel Growth Factor That Maintains Human Stem Cells in a More Naïve State
Source: PLoS One. 2013 Mar 7;8(3):e58601. doi: 10.1371/journal.pone.0058601 (PMC3591366; doi:10.1371/journal.pone.0058601)
Supplement: Methods S1 — Supplementary Methods. (DOC) [file pone.0058601.s013.doc]

**Supplementary Methods**

***Protein oligomarization state.***

NM23-wt, NM23_S120G_-hexamer, NM23_S120G_-mixed (50% dimer) and NM23_S120G_-dimer (5 and 10 µg/well) were subjected to a blue native gel (NativePAGE™ Novex® 4-16% Bis-Tris, Invitrogen) following the manufacturer instructions and the gel was stained with coomassie blue.

NM23-wt, NM23_S120G_-hexamer and NM23_S120G_-dimer (32nM, 16nM and 8nM) were subjected to a blue native gel (NativePAGE™ Novex® 4-16% Bis-Tris, Life Technologies) following the manufacturer instructions and transferred to a polyvinylidene difluoride (PVDF) membrane (Millipore) for western blot.

***NM23 western blot***

Membranes were washed 3 times for 10 min in PBST (PBS pH 7.4 + 0.05% Tween20) and probed with anti NM23-H1 antibody (C20, Santa Cruz Biotechnology. 1/200 dilution) overnight at 4°C, followed by washing (3 time 15 min in PBST) and horseradish peroxidase-conjugated rabbit anti-IgG (Pierce. 1/12500 dilution) for 1 h at room temperature. Antibodies were diluted in PBST containing 5% milk. Detection was with Immun-Star Substrate (Bio-Rad) or the SuperSignal West Femto Chemiluminescent Substrate (Pierce).

***NM23 degradation***

NM23_S120G_-dimer was diluted in DMEM/F12/GlutaMAX, 20% Knock out Serum Replacement, 1% Non-essential Amino Acid 0.1mM β-mercaptoethanol to a concentration of 16nM. An aliquot (100µL) for each time point was incubated at 37°C and the aliquot was taken out and stored at 4°C after 1, 2, 4, 8, 24 and 48h. 20µL of each aliquots were subjected to a reducing SDS-PAGE and transferred to a polyvinylidene difluoride (PVDF) membrane (Millipore) for western blot.

***Karyotype Analysis of ES Cells***

Exponentially growing cultures of cells used in experiments described were prepared in T-25 flasks coated with matrigel. Karyotype analysis was performed by Cell Line Genetics (Madison, WI).

***Flow cytometry analysis***

FACS analysis was performed using HES-3 stem cells (Biotime Inc. Alameda, CA) grown on a anti-MUC1* antibody coated surface, dissociated using 0.05% trypsin-EDTA (Life Technologies #25300-120) and resuspended in 100 ul PBS + 1% BSA. HES-3 stem cells were stained for markers of pluripotency including Tra1-60 (DyLight-488, 1:100; Stemgent Cat # 09-0068), PE-conjugated SSEA-4 (5 ul per sample, BD Biosciences, Cat.# 560128) and APC-conjugated SSEA-3 (5 ul per sample, BD Biosciences, Cat.# 561145) as well as a marker for differentiation, CXCR4 (1:100, eBioscience, Cat#. 14-9999-82). Stained cells were analyzed on a BD LSR II flow cytometer using FACSDiva software (BD Biosciences) at the Dana Farber Cancer Institute Flow Cytometer Core (Jimmy Fund).

***Embryoid body formation***

Cell from a confluent well were dissected, washed, resuspended in NM3-MM without growth factor (4mL) and plated in ultra low attachment plate (Costar #3471). The media was changed after 3 and 5 days. The EBs were then collected, washed, resuspended in NM23-MM without growth factor and plated (400uL/chamber, 10-15 EBs) on gelatin coated permanox 4 well chamber slides (lab-Tek #177437). After 2 days EBs were feed 2-3 weeks until all EBs are spread in a monolayer with different cell types.

***Quantification of Immunofluorescence images***

Immunofluorescence data for embryoid body formation (Fig 3c-h) was quantified using the NIH image analysis program Image J (National Institutes of Health, Bethesda, Maryland, USA). Images were split to their respective channels and the blue channel representing DAPI nuclear staining was used for total cell counting using the Cell Counter macro. Cells positive for one of the differentiation markers (nestin, B-tubulin, smooth muscle actin or alpha feta protein) were quantified using the same Cell Counter macro and a percentage of positive cells versus total number of cells was calculated. At least 4 separate images were used per measurement.

***Statistical analysis***

We considered passage number and expression as continuous variables.  We performed a log-transformation on the gene expression responses to meet the linear models' distributional assumptions.  After taking logarithms, the responses were roughly normally distributed.  Our linear models included passage number as a covariate and primed/naïve and individual genes as fixed effects.  We specified genes as a nested effect within primed or naïve.  We also modeled explicitly the correlation between KLF4 and NANOG. 
In our results, our estimated means from the general linear model are least squared estimates.  All correlation results are Pearson correlations.  Multiple comparisons of means are from a sequential testing of ANOVA followed by Fisher's least significant difference p-values.
